# Assessing barriers to ecosystem-based adaptation: Application to tropical islands

**DOI:** 10.1017/cft.2026.10035

**Published:** 2026-06-03

**Authors:** Virginie, K.E. Duvat

**Affiliations:** 1 LIttoral ENvironnement et Sociétés (UMR LIENSs 7266), La Rochelle University-CNRS, France; 2 French University Institute (IUF), France; 3 Cawthron Institute, New Zealand

**Keywords:** climate change, barriers and limits to adaptation, assessment methods, ecosystem-based adaptation, France

## Abstract

Barriers to ecosystem-based adaptation (EbA), which is part of the broader nature-based solutions (NbS) category, remain poorly understood and assessed. This article addresses this research gap, by proposing a structured analytical framework to understand barriers and applying it to 24 coastal EbA projects deployed in French tropical island territories. This framework considers four dimensions of barriers: categories, origins, impacts on the adaptation process, and temporalities. The findings highlight three main barriers relating to institutions, governance, politics, laws and regulations (35%); awareness, knowledge and technical resources (20%); and finance (15%). Most barriers are objective (80%), contextual (51.7%) and not adaptation specific (63.3%). Prevalent adaptation-specific barriers are the lack of or weakness of EbA-oriented policy and tools (59.5%) and lack of a future-oriented risk- and solution-based approach (27.0%). Most barriers (56.7%) affect two stages of the adaptation process (readiness and implementation) and were not overcome over the lifetime of the projects (53.3%). Thirteen solutions to barriers were implemented, with information, knowledge and awareness sharing or strengthening and increased coordination efforts being the most utilised and successful. This study highlights the benefits of including a barrier-oriented analysis in the evaluation of adaptation projects and proposes an operational and transferable framework to do so.

## Graphical abstract

**Figure:**
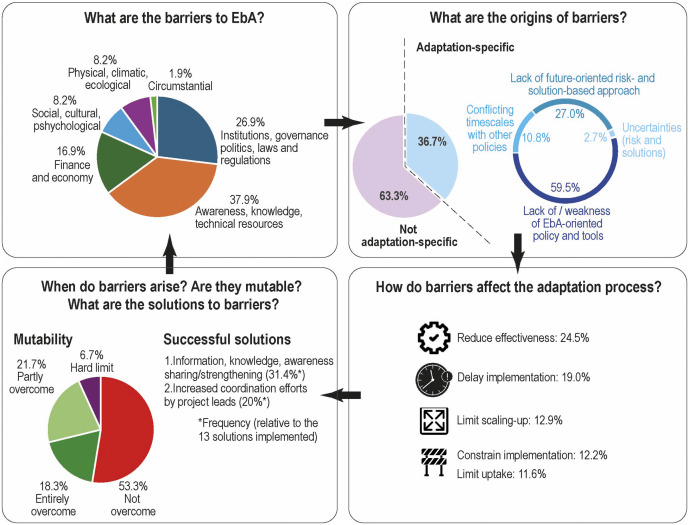

## Impact statement

First, this article proposes a structured and transferable analytical framework to describe and assess the barriers to ecosystem-based adaptation (EbA), as part of broader nature-based solutions (NbS). This analytical framework addresses two major gaps highlighted by scholars and practitioners, respectively the lack of understanding and the lack of assessment of adaptation barriers. It comprises four dimensions combined in an iterative process enabling the monitoring and final assessment of the barriers arising during an adaptation process. These dimensions are the nature of barriers (categories and subcategories), their origins (objective versus subjective, level of occurrence and adaptation specificity), their impacts on the adaptation process and their temporalities.

Second, this article applies this analytical framework to 24 EbA projects deployed in French tropical island territories of the Caribbean and Indian and Pacific Oceans. Thus, it demonstrates its operationality and provides unprecedented insights on the barriers to EbA in these territories. It first shows that 37.9% of barriers relate to risk- and EbA-related awareness, knowledge and technical resources, whereas 26.9% of barriers relate to institutions, governance, politics, laws and regulations. These root barriers create or generate other barriers (e.g. limited EbA funding) through path dependency involving both objective and subjective factors. Moreover, 63.3% of barriers are not adaptation specific, meaning that removing them would strengthen public policies of any kind. The three main impacts of barriers are the reduction of EbA effectiveness, delay in implementation and limitation of upscaling. More than 53% of barriers were not overcome during the lifetime of the EbA projects. Sharing and strengthening knowledge and reinforcing coordination efforts were the two most used and most effective solutions to barriers.

Third, the lessons learnt from the 24 projects analysed provide useful guidance for designing more robust EbA projects and call for the systematic inclusion of in-depth barrier analysis in EbA and other adaptation projects.

## Introduction

In the 2000s, it became clear that the degree of implementation and success of climate change adaptation policies, including ecosystem-based approaches, were determined by context-specific barriers and levers (Adger et al., 2007). Following the Fourth IPCC Assessment Report (AR4), this observation led scholars to call for the prioritisation of the analysis of barriers to better support adaptation progress. Research efforts on barriers to adaptation then took three directions: (1) clarifying concepts related to barriers and limits (see Box 1 for the definitions of the key concepts used in this study); (2) identifying barriers and categorising them, as related to (i) institutions, governance, politics and laws and regulations; (ii) awareness, knowledge and technical resources; (iii) finance and economy; (iv) social, cultural and psychological factors; (v) physical, climatic and ecological factors; and (vi) circumstantial factors, for example, the COVID-19 pandemic or a political crisis (Klein et al., 2014; Mycoo et al., 2022; Rahman et al., 2023; Malik and Ford, 2025); and (3) determining promising avenues for investigating them, by distinguishing between proximate and root (or deep) barriers and between objective and subjective barriers; tracing their origin (actor-, system-, or context-related) to help remove them; identifying adaptation-specific barriers; determining whether they relate to the adaptation process or its outcomes; and finding ways to overcome them (Moser and Ekstrom, 2010; Sutton and Tobin, 2011; Biesbroek et al., 2013; Eisenack et al., 2014; Barnett et al., 2015; Kim and Shin, 2024).

BOX 1. Definitions of key concepts.

Definitions of the key concepts used in this study.

**Adaptive performance:**

Describes the extent to which an EbA project meets climate change adaptation requirements; is based upon eight variables, including the implementation context; governance; funding; social acceptability of the EbA; its risk reduction potential; support by studies, monitoring and evaluation protocols; the cobenefits and disbenefits generated; and its overall contribution to adaptation at a broader scale (see Duvat et al., 2025 for details).

**Barriers to adaptation:**

***Barrier*** (also referred to as *challenge, constraint, limit*, and *problem*; Klein et al., 2014; Lee et al., 2022) is a “*Factor* [s or processes] *that make it harder to plan and implement adaptation actions* [including Ecosystem-based]” and thereby *“restrict the variety and effectiveness of options for actors to secure their existing objectives, or for a natural system to change in ways that maintain productivity or functioning”* (Klein et al., 2014: 907). *“A barrier to adaptation can be overcome with concrete efforts, creative management, new ways of thinking, prioritization, and changes in resources, land uses, institutions*, etc.*”* (Lee et al., 2022: 2).

***Lever*** (also referred to as *opportunity, enabler* or *enabling condition*, or *leverage)*: It refers to a facilitator of adaptation action planning and implementation (Lee et al., 2022)

***Deep* or *root barrier:*** It is a barrier that has strong historical roots, for example, local leadership or stable support from higher governance levels (Klein et al., 2014). Root barriers influence and generally create or aggravate other barriers and are trapped in self-reinforcing path dependency (Barnett et al., 2015; Rahman et al., 2023). Examples include institutional barriers, the lack of communication and resources, the low priority of climate change adaptation and cultural values (Lee et al., 2022; Dubo et al., 2023; Rahman et al., 2023).

***Objective barrier:*** It is a factor that reduces the ability of an individual or institution to implement adaptation. Examples include the lack of time or unfavourable regulatory tools (Sutton and Tobin, 2011).

***Proximate barrier***: It refers to apparent or immediate conditions impeding adaptation that are linked to deeper barriers. Examples include unresolved conflicts between involved actors (Eisenack et al., 2014) or unfavourable regulations (Moser and Ekstrom, 2010).

***Subjective barrier:*** It refers to “*perceptions of reality”*, that is, cognitive or affective factors limiting the desire of an actor or institution to become engaged in adaptation (Tanner, 1999). Examples include administrative procedures experienced as cumbersome, multiple partnership experienced as complex or the strong sense of place of residents.

**Limits to adaptation:**

***Limit:** “The point at which an actor’s objectives or system’s needs cannot be secured from intolerable risks through adaptive actions”* (Klein et al., 2014: 907).

***Hard limit:*** *“When communities, species and ecosystems cannot adjust to new climate regimes in time to avoid degradation or collapse”* (Barnett et al., 2015: 2). “[When] *no further autonomous or planned adaptive actions are possible to avoid intolerable risks*” (Juhola et al., 2024: 1). Examples in ecological systems include coral reef decline (Barnett et al., 2015) or species range loss (Juhola et al., 2024). Examples in human systems include physical limit to relocation (Barnett et al., 2015), for example, in atoll settings or heat thresholds for human health (Juhola et al., 2024).

***Soft limit:*** *“Options are currently not available to avoid intolerable risks through adaptive action”* (Klein et al., 2014: 907). These are socially determined, dynamic and contingent constraints (Barnett et al., 2015) that may be overcome in the future because they are mutable (Juhola et al., 2024).

This body of literature provides a useful theoretical foundation for the analysis of barriers to adaptation. However, our understanding of barriers remains very general and limited mainly to their categorisation (e.g. IPCC AR6) in the absence of a structured analytical framework for analysing them in detail and developing case studies. This is particularly true in the field of ecosystem-based adaptation (EbA) that constitutes an emerging, increasingly recognised and funded, category of adaptation measure across temperate and tropical regions (Chausson et al., 2020). The EbA literature, which falls more broadly within the field of nature-based solutions (NbS), has grown considerably in recent years. It mainly focuses on (1) defining NbS and EbA, from fully natural to hybrid solutions (Pontee et al., 2016; Seddon et al., 2020a); (2) highlighting their potential in reducing climate risk and being cost-effective (Duarte et al., 2013; Feagin et al., 2015; Morris et al., 2018; Logan et al., 2018; Cohen-Shacham et al., 2019; Beck et al., 2022; Vicarelli et al., 2024; Twomey et al., 2025); and (3) discussing, generally without drawing on specific case studies, the constrains and limits they face (Temmerman et al., 2013; Nalau et al., 2018; Barnett et al., 2022; Johnson et al., 2022). Most EbA and NbS studies have thus remained rather theoretical. Moreover, they focus on urban settings and the Global North (Chausson et al., 2020; Ruangpan et al., 2020; Goodwin et al., 2024). Tropical islands have been the subject of very few studies. Those studies have focused on small island developing states (SIDSs), ignoring the overseas territories of developed countries, and on terrestrial ecosystems, overlooking coastal ecosystems (Chausson et al., 2020; Brown et al., 2026). As a result, we still have a very limited understanding of coastal EbA and the barriers they face in tropical islands despite the recognition that EbA is increasingly used in tropical islands (e.g. in Oceania) and could play a major role in supporting adaptation in these settings (Hills et al., 2013; Ferrario et al., 2014; Giffin et al., 2020).

This article addresses these research gaps by advancing knowledge on barriers to coastal EbA in French tropical island territories through a twofold approach. First, it proposes a new and transferable analytical framework aimed at supporting in-depth assessments of barriers to EbA, and adaptation more broadly. This research framework builds on available theoretical literature and addresses four main questions: (1) What are the main barriers to EbA? (2) What are their origins? (3) How do barriers affect the adaptation process? and (4) What are their timeframes? Second, this article applies this analytical framework to 24 coastal EbA projects deployed in French tropical islands of the Caribbean region and Indian and Pacific Oceans. It thus makes methodological and cognitive contributions to the current state of knowledge on barriers to EbA in tropical islands. It highlights the specific root barriers to EbA operating in these islands, as well as the vicious circle effects they generate, and proposes concrete ways to overcome them.

## Context of the study

### Details of the studied coastal EbA projects

**Figure 1.**
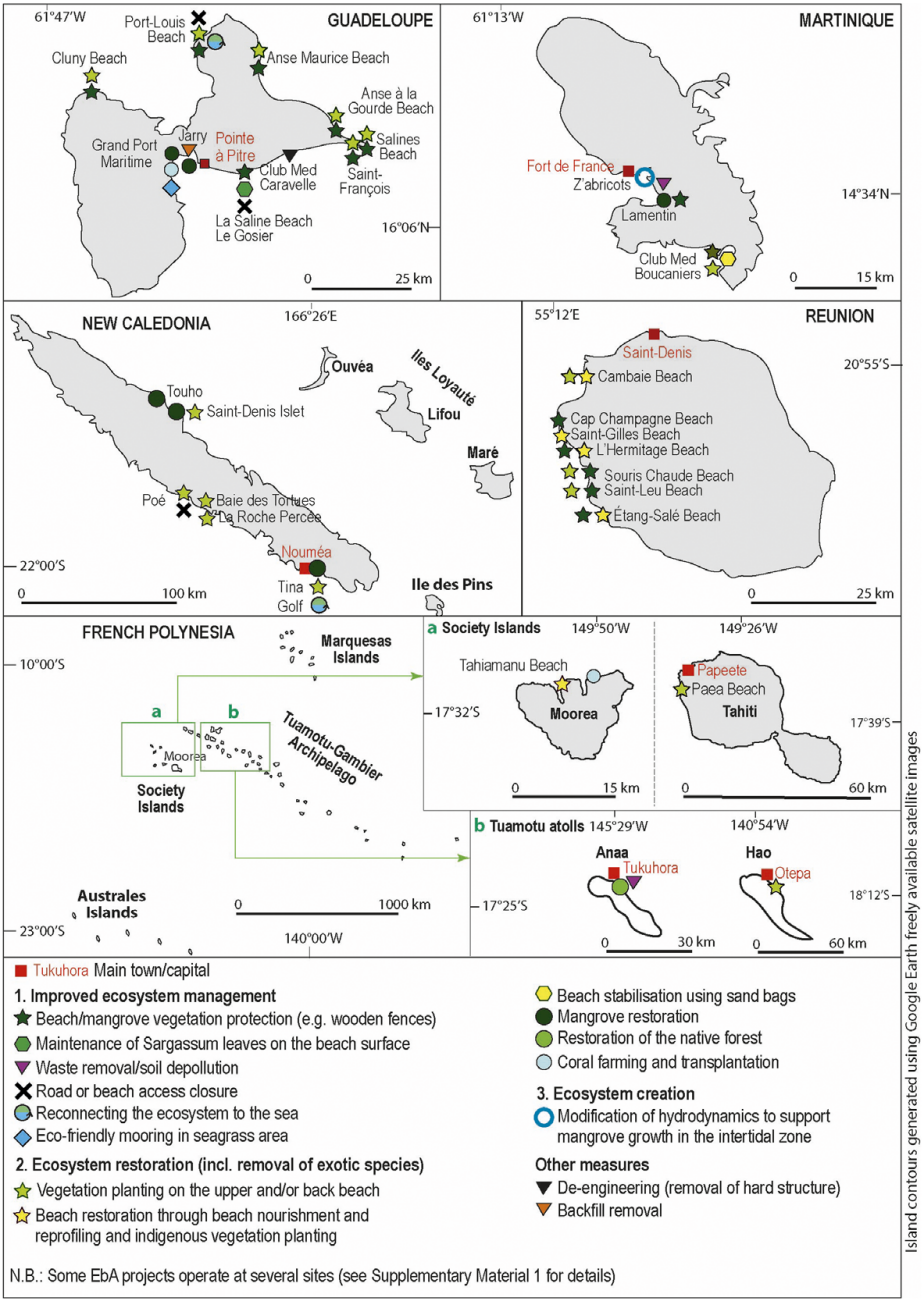
Mapping of studied ecosystem-based adaptation (EbA) projects with the specific type of intervention undertaken. These maps highlight the location and nature of EbA projects with the various interventions implemented (refer to icon key for interventions) in the five study French overseas tropical island territories. Projects are classified by EbA type (1–3 in the legend), and technical actions are described. Figure 1. long description.

**Figure 2.**
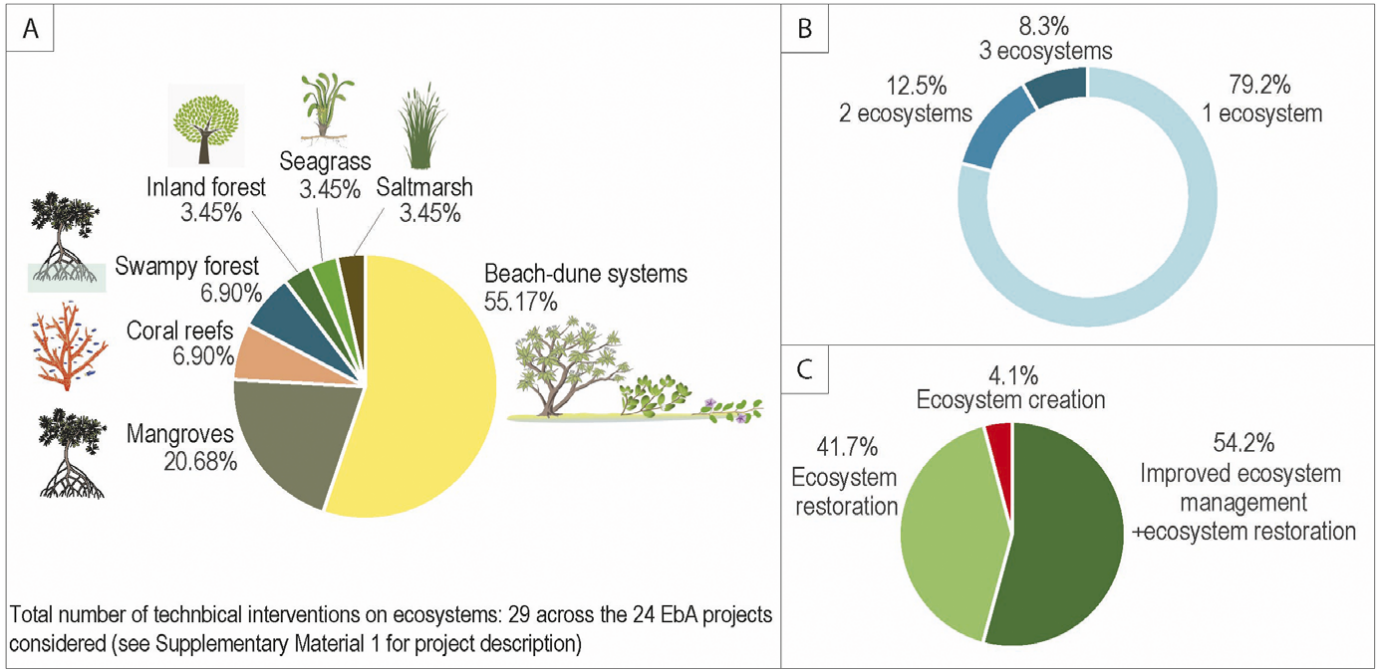
Main characteristics of studied EbA projects. Panel A shows the ecosystems targeted by the 24 coastal EbA projects of interest (see the Supplementary Material 1 and Figure 1 for project description and location, respectively). It highlights the prevalence of EbA projects targeting beach-dune systems and mangroves. Panel B emphasises the number of ecosystems (1, 2 or 3) considered by these projects. Panel C shows the types of technical interventions deployed under these projects, indicating the prevalence of improved ecosystem management and ecosystem restoration. Figure 2. long description.

This study considered 24 coastal EbA projects distributed across five French tropical island territories: Martinique (3 projects; P1–P3 in the Supplementary Material 1 that provides details about the projects) and Guadeloupe (7; P4–P10; Supplementary Material 1) in the Caribbean region; Reunion Island in the Indian Ocean (5; P11–P15; Supplementary Material 1); and French Polynesia (5; P16–P20; Supplementary Material 1) and New Caledonia (4; P21–P24; Supplementary Material 1) in the Pacific Ocean (see Figure 1 for locations of the projects). These projects were all aimed at reducing coastal risks, namely coastal erosion (17 projects), marine flooding (4) or both risks (3; Supplementary Material 1). Some of them operate at several sites. They reflect a variety of situations, as 41.7% of them were deployed in natural or rural areas, 37.5% in peri-urban areas, and 20.8% in urban or industrial areas. They involve a wide range of ecosystems, actions and actors (Figure 2). Project leads were primarily institutional actors operating at the local and national levels (13 projects out of 24) and local associations (7/24), with these two categories of actors jointly implementing an additional project (1/24). Private actors, including tourism companies (2 projects) and the Great Harbour of Guadeloupe (1 project), also led projects. Except for one project which started in the 2000s, EbA projects were initiated between 2010s and 2020s, with six projects out of 24 still ongoing today. They were more or less successful in addressing climate change challenges. The assessment of their adaptive performance (see Box 1 for definition) highlighted contrasting performances, with synthetic indices ranging from 39.4% (low performance) to 77.2% (high performance) (Duvat et al., 2025).

### Methods

This study relies on a three-step approach comprising the design of a barrier-oriented analytical framework, a data collection phase and a data treatment and analysis phase.

#### A new analytical framework for the understanding of adaptation barriers

**Figure 3.**
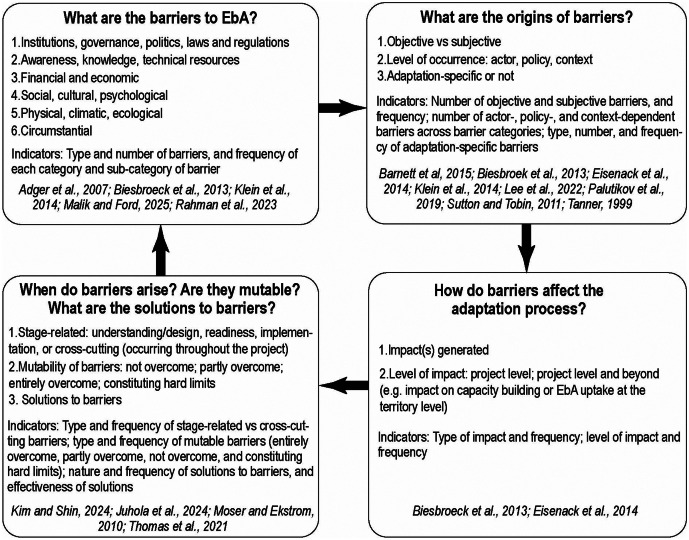
A new analytical framework for the understanding of barriers to adaptation.This framework addresses the research gaps highlighted in the scientific literature (see references in boxes), using a semi-quantitative approach. Four interrelated dimensions are considered: the nature, origins, impacts on the adaptation process and temporalities of the barriers. Addressing them enables better understanding of barriers and the identification of solutions to overcome them. This framework constitutes an iterative approach (shown by arrows in the figure) aimed at supporting adaptation progress. Figure 3. long description.

This study proposes a new analytical framework investigating four interrelated dimensions of barriers: their nature, origins, impacts on the adaptation process and temporalities (Figure 3). This framework builds upon previous scientific studies and provides a structured and in-depth approach for the understanding of barriers to adaptation. In this study, this analytical framework is applied to EbA.

First (top-left box in Figure 3), in line with previous studies, especially IPCC work dating back to the AR4 (Adger et al., 2007) as well as complementary analyses (e.g. Rahman et al., 2023), barriers were classified into six main categories. These categories reflect both proximate barriers corresponding to immediate and factual obstacles to EbA (e.g. lack of funding, or circumstantial factors such as the COVID-19 crisis) and deep or root barriers pointing to powerful underlying obstacles to EbA, such as historically weak institutions or the lower priority given to climate change adaptation compared to development issues (Barnett et al., 2015; Lee et al., 2022). Because they are deeply enrooted in history and therefore trapped in self-reinforcing path-dependency, root barriers are the hardest to overcome. They also influence proximate barriers: for example, the low priority given to adaptation (root barrier) generally explains the limited funding of EbA (proximate barrier). This is precisely because they constitute strong obstacles to adaptation and key controls over the whole adaptation process that they must be identified and overcome. This first step of the analysis facilitates describing the main categories and sub-categories of barriers and quantifying their relative weight.

Second (top-right box in Figure 3), the origins of barriers were investigated through a three-step approach interrogating their objective versus subjective character, level of occurrence and adaptation-specificity. The number of objective and subjective barriers and their frequency, defined as the number of projects facing a barrier, were calculated. In addition, the level of occurrence of barriers was determined considering that barriers can emerge at three levels: actor level, as “*what actors value as barriers depends on their roles, values, interests, and ideas*” (Biesbroek et al., 2013: 1124); policy or governance level, that is, within the process of developing and implementing EbA; and local-to-international context level, that is, within the system in which EbA takes place (Biesbroek et al., 2013; Barnett et al., 2015). The frequency of actor-, policy- and context-dependent barriers across the various categories and sub-categories of barriers was determined. Finally, adaptation-specific barriers were identified and their frequency analysed. This enabled exploration of whether the adaptation-specific barriers described in the literature, including conflicting timescales between well-established short-term policies and actions and the long-term impacts of climate change; persistent uncertainties about the nature and scale of climate risk and effectiveness of available adaptation measures; and institutional fragmentation conflicting with the cross-sector, cross-policy, and cross-scale nature of adaptation (Eisenack et al., 2014), applied to EbA.

Third (bottom-right box in Figure 3), the impacts of barriers on the adaptation process (e.g. delays in action) were described and their frequency was calculated for each type of impact. This analysis was conducted at the project and territory levels, with the aim of documenting the scale of the impacts.

The fourth step (bottom-left box in Figure 3) interrogated the temporalities of barriers, firstly, by questioning whether the barriers affected a specific stage of the EbA process (e.g. readiness) or were cross-cutting and impacting the whole process. In line with Kim and Shin (2024), three stages are distinguished in this study, depending on whether the barriers relate to the understanding/design stage (*are stakeholders aware of present-to-future climate risk and associated EbA-specific requirements?*), the readiness stage (*are stakeholders well-prepared and having the capacity to deploy the EbA project, based upon risk information, size of staff, legislation*, etc.*?)* or the implementation stage (*are stakeholders able to deploy successfully the EbA project on the ground?*). Secondly, acknowledging that barriers are social constructs that can be overcome by increased efforts and transformational changes (Barnett et al., 2015; Thomas et al., 2021; Juhola et al., 2024), we analysed their mutability over the lifetime of projects. To this end, barriers were classified as not overcome, partly overcome, entirely overcome or constituting hard limits to EbA. For those barriers that were partly or entirely overcome, implemented solutions and their success were analysed.

#### Documenting barriers

EbA projects were documented using technical reports, semi-structured interviews and field visits with project leads and their partners, and on-site final workshops aimed at sharing and discussing the results generated. In line with actor-centred approaches addressing barriers to adaptation (e.g. Kim and Shin, 2024; Twomey et al., 2025), interviews provided most of the material collected at the project level.

The interview guide comprised sections on objectives, origin and duration of projects; ecosystem(s) targeted; nature of action(s); context (institutions, policies and regulations, land tenure); spatial scale of action(s); capacities (staff and skills, coordination and awareness-raising, technical support, knowledge, monitoring and evaluation); funding; technical effectiveness; social acceptability and support; co-benefits and disbenefits; levers and barriers to the design, implementation and success of projects; solutions to overcome barriers and outcomes (see details in the Supplementary Material 2). Levers and barriers were first collected at the project level during interviews and field visits and then discussed collectively during final on-site workshops with project leads and partners. The workshops allowed cross-checking of the occurrence of barriers across EbA projects, refining the analysis of barriers, and identifying collectively solutions to overcome the barriers.

#### Data treatment and analysis

The data collected on barriers were integrated into two complementary databases. The first database provides the full description of the 60 inventoried barriers, using the categorisation (1, 2, 3, etc.) described in Figure 3, and lists the EbA projects facing each barrier. This database constitutes Supplementary Material 3 and provides illustrative examples. It served as the basis to build the second database (Excel file), which contains the application of the analytical framework to the 60 barriers. This second database, which constitutes Supplementary Material 4, reuses the barrier numbering system used in the Supplementary Material 3. It therefore contains four tabs corresponding to the four dimensions of barriers investigated (type, origins, impacts and temporalities). For each of them, the frequency of occurrence was calculated. In the Results section, this semi-quantitative analysis is illustrated using frequency graphs aimed at providing an overview of the results generated. It is supplemented by a qualitative analysis, using concrete examples extracted from the Supplementary Material 1 (description of barriers) and quotes extracted from interviews and workshops. Supplementary Materials 3 and 4 files thus form the basis of this study.

## Results

The results are presented following the four steps of the analytical framework.

### Categorisation of barriers to EbA

**Figure 4.**
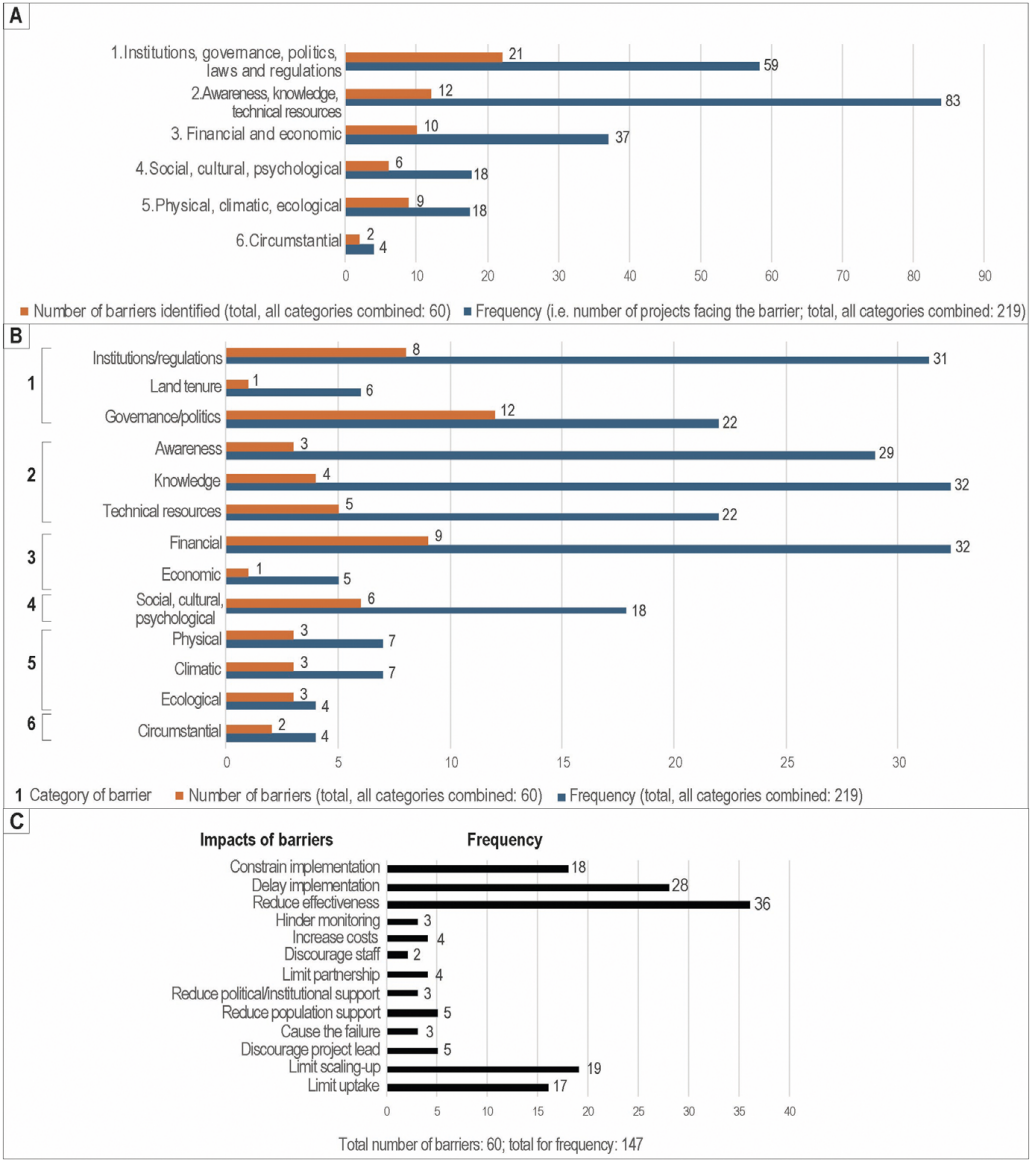
Distribution of barriers per category and sub-category, and impacts of barriers on the adaptation process. Panel A highlights the number of barriers identified for each category (e.g. 2 barriers for circumstantial factors, for example, the COVID-19 pandemic) and their frequency (number of projects facing the barrier). Panel B details the results obtained for each sub-category of barrier. Panel C shows the 13 types of impacts of barriers on the adaptation process that arose from the 24 projects analysed, and their frequency. Figure 4. long description.

Sixty barriers were identified across the 24 EbA projects analysed that were grouped into the six main categories described in Figure 3 (Figure 4A,B; see details in the Supplementary Material 3). The three categories exhibiting the highest numbers of barriers and highest frequencies (i.e. highest numbers of projects facing the barrier) were (1) institutions, governance, politics and laws and regulations (21 barriers out of 60; 59 occurrences out of 219); (2) awareness, knowledge and technical resources (12/60; 83/219); and (3) finance (9/60; 32/219) and economy (1/60; 5/219) (Figure 4A,B). These categories mainly include root barriers involving path dependency. The first root barrier is the weakness of institutions, including the lack of institution in charge of EbA (Projects P16, P18 and P19, French Polynesia; see the Supplementary Material 1 for project description and the Supplementary Material 3 for barrier description) and a lack of regulatory tools to support EbA implementation (P20; French Polynesia); the limited human capacity devoted to EbA (P8, Guadeloupe; P16 and P20, French Polynesia); and the limited political support to EbA (P4, Guadeloupe; P20, French Polynesia). Other root barriers are the lack of awareness and knowledge about EbA specificities as an adaptation policy (all projects) and about the protection service delivered by coastal vegetated systems and mangroves among elected representatives and the population (P5 and P6, Guadeloupe; P22, New Caledonia); the lack of technical resources, such as expertise (10 projects, see details in the Supplementary Material 3) and equipment (7 Projects; Supplementary Material 3) to support EbA; and inappropriate financial tools and rules (10 projects; Supplementary Material 3).

These three dominant categories of barriers are followed firstly, by physical (e.g. limited site accessibility, P14; limited space for EbA deployment, P20), climatic (flood damage, heavy rainfall and drought; 6 projects; see details in the Supplementary Material 3), and ecological (e.g. predation, P21; degraded ecological conditions, P9 and P17) barriers (9 barriers out of 60; 18 occurrences out of 219); and secondly, by social (e.g. vandalism, 6 projects; Supplementary Material 3), cultural (beach “cleaning culture’” that is, coastal vegetation removal, in Guadeloupe and New Caledonia, P5, P6 and P22) and psychological (strong sense of place and attachment of users to exotic tree species, P12, Reunion) barriers (6/60; 18/219; Supplementary Material 3).

All categories combined, the five main barriers faced by project leads and partners are (1) a lack of awareness and knowledge about the specific requirements of EbA as an adaptation policy compared to conventional ecosystem restoration and management. This mainly includes the spatial scale (i.e. landscape or geomorphic unit scale) and temporal scales (i.e. lead time until full effectiveness and duration of risk reduction benefits under climate change) to be considered, as well as the need for risk assessment and monitoring, and for EbA maintenance and evaluation. These shortcomings apply to all 24 projects. During workshops, most project leads acknowledged that they had applied the conventional restoration and management approaches they were accustomed to using and had therefore overlooked the specific requirements of EbA as an adaptation policy. Even the most robust technical-scientific teams confessed that they lacked a baseline against which to measure the effectiveness of the EbA in reducing coastal erosion and/or marine flooding.

(2) the cumbersome nature of national administrative and regulatory procedures (10 projects; Supplementary Material 3), especially related to public procurement, technical interventions on the maritime public domain and handling of protected coral and plant species. Regarding the last point, the lead of Project 11 (coastal vegetation restoration, Reunion; Supplementary Material 1) explained “:*The regulation protecting rare species places significant constraints on the choice of species we can plant (…). The requirement for genetic traceability of seedlings greatly limits the possibilities for diversifying the plant palette (…). When working on projects that are meant to promote biodiversity, this is a major obstacle”.* The project team adapted to this constraint by selecting only authorised plant species, which reduced plant diversity and potentially young plants’ survival rate. Moreover, project leads explained that French public procurement rules were much restrictive overseas due to smaller staff sizes and lower levels of expertise resulting notably from high turnover.

(3) a lack of technical resources at project or territory level (10 projects; Supplementary Material 3), including nurseries for the production of native plant species (P16 and P19; French Polynesia), local expertise for soil decontamination in industrial areas (P8 and P9; Guadeloupe) and specialised machinery (e.g. spider excavator to remove invasive plant species, P11; Reunion).

(4) inappropriateness of multi-year funding tools for EbA (9 projects). For example, the lead of Project 4 (multi-site coastal vegetation restoration; Supplementary Material 1) explained: “*We had initially planned to document the impact of vegetation restoration enclosures on coastal erosion. But by the time the enclosures are installed and the vegetation has grown, even if we had had baseline data, we wouldn’t have had enough time over the three years of the project to assess whether this measure reduces erosion”.*

(5) limited implementation of regulations related to coastal development and (waste)water management, acting as a barrier to the success of restoration actions, especially in Caribbean and Pacific islands (8 projects; Supplementary Material 3), for example, the lead of Project 6 (soft coastline management, Guadeloupe; Supplementary Material 1) confessed: “*This must be a team effort, and everyone needs to play their part […]. You can invest money and be highly committed to your part, but if other stressors such as wastewater discharges aren’t stopped, restoration may fail”.*

### Origins of barriers

**Figure 5.**
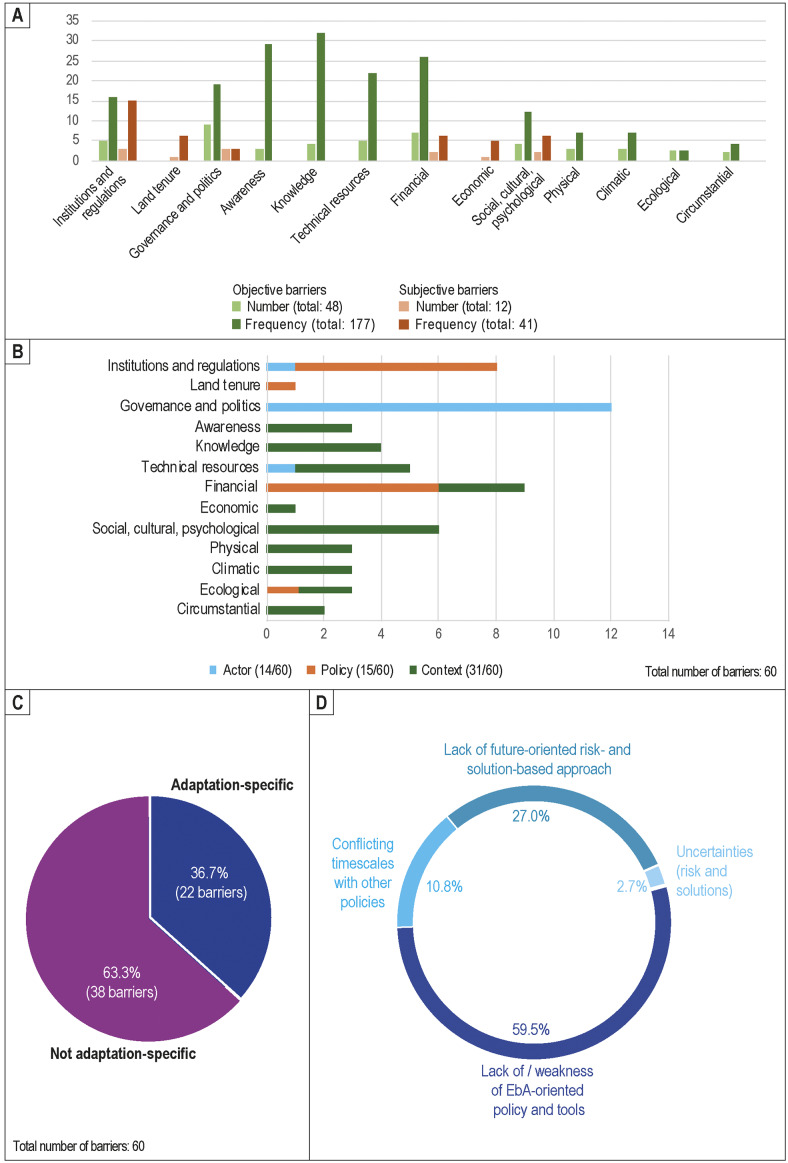
Origins of barriers to EbA. Panel A shows the distribution of barriers according to their objective versus subjective nature. Panel B emphasises the three levels, namely actor, policy and context, at which barriers arise and highlights their relative frequency. Panels C and D highlight that most barriers are not adaptation specific (Panel C) and detail adaptation-specific barriers (type and frequency). Figure 5. long description.

Among the 60 barriers identified, respectively 80% (48) and 20% (12) relate to objective and subjective factors (Figure 5A). Objective barriers mainly involve governance and politics, especially the lack of political support, evidenced by the lack of involvement of mayors in projects, and of population support, evidenced by low participation in participatory activities, occurring in all territories; limited stakeholder involvement in EbA (4 projects; Supplementary Material 3); and differences of values and management practices creating delays in implementation and conflicts between local and national institutional actors and between institutional actors and the population (P4, P6 and P12; Supplementary Material 3). For example, in Guadeloupe, several projects faced misunderstandings, tensions and conflicts arising from the confrontation of different visions and practices of restoration. At the national level, the French Coastal Conservatory (FCC) and National Forestry Office (NFO) have two different visions of what a natural environment is and of how to restore it. While the FCC works with nature by maintaining or re-establishing the processes ensuring the regeneration of ecosystems, the NFO follows a more interventionist approach involving plant regeneration enclosures and soil decompaction. This approach requires specific equipment (e.g. watering systems) and is more visible to beach users and can therefore cause public opposition. At a second level, differences of visions and practices also exist between national and local institutional actors. Still in Guadeloupe, a vegetation restoration project aimed at reducing beach erosion (P4 in the Supplementary Material 1) faced a conflict first, with the mayor and technical services of a municipality over the maintenance techniques to be used (mechanical or manual), and second with beach users rejecting restrictions on motor vehicle access to the beach. Another objective barrier is finance, especially the inappropriateness of multi-year projects that are too short for ensuring successful EbA, difficulties in raising funds (7 projects in the Caribbean and Pacific; Supplementary Material 3) and restrictive funding rules and budgets preventing the recruitment of skilled staff or the carrying out of some tasks (6 projects; all ocean basins; Supplementary Material 3). As explained by a project lead (P9, Guadeloupe, coral restoration), compounding barriers related to governance and funding rules hinder action: “*In European LIFE projects, external services must not exceed 35% of the total budget. Since the director general* [of the lead structure of the project] *refused to allow the project team to conduct the monitoring dives, we are now faced with two combined constraints that prevent us from carrying out this work*”. The third and fourth objective factors, already mentioned above, relate to institutions (limited staff for EbA) and regulations (lack of implementation of waste and wastewater management regulations) and to a lack of technical resources. Subjective barriers also mainly relate to institutions and regulations and include the experience of national and European administrative and regulatory procedures applying to EbA as cumbersome, as well as the perception of multi-partner project design and implementation as complex.

The analysis of the level of occurrence of barriers (Figure 5B) reveals that barriers predominantly relate, firstly, to the local context (51.7%), characterised by a lack of awareness, knowledge, technical resources and finance, and secondly to constraining physical, climatic, and ecological conditions. Other barriers emerge at policy (25%; institutions and regulations and finance) and actor (23.3%; governance and politics) levels.

Twenty-two barriers out of 60 (36.7%) are adaptation specific (Figure 5C), including the lack of or weakness of EbA-oriented policy and tools (22 occurrences out of 37, representing 59.5% of adaptation-specific barriers), the lack of future-oriented risk- and solution-based approach (10/37; 27.0%), conflicting timescales between EbA and other public policies (4/37; 10.8%), and uncertainties related to future climate risk and solution effectiveness (1/37; 2.7%) (Figure 5D).

### Impacts of barriers on the EbA process

This study identified 13 different negative impacts of barriers on the EbA process (Figure 4C). The five predominant impacts are, in order of importance, reduced effectiveness (36 occurrences out of 147); delay in implementation (28/147); limitation to upscaling through the expansion of the project area or transfer of the project to other sites (19/147); difficulties in implementation (18/147); and a reduction of EbA uptake at the territory scale (17/147). Barriers reduced the potential effectiveness of projects by generating high mortality rates of restored species and a waste of time and resources, which in turn reduced the size of restored areas and their potential to reduce risk. Examples are the destruction of restored mangrove areas by river flooding, low survival rates of transplanted corals in polluted areas, high mortality rates of young plants due to the absence or inadequacy of watering systems or to vandalism and the absence of preliminary studies leading to the choice of ineffective restoration techniques (Supplementary Material 3).

Other impacts faced by EbA projects 2 to 5 include the failure of two coral restoration projects (P9 in Guadeloupe; and P17 in French Polynesia; Supplementary Material 1), hindrance to monitoring, increased costs, discouragement of EbA project holder and staff, limitation of partnership and reduced political and/or public support (Supplementary Material 3).

Some barriers had cumulative impacts. For example, some project leads explained that the lack of awareness of local institutions about the coastal protection service provided by vegetated systems led to mechanical cleaning interventions that caused high mortality rates of plants in restored beach areas, limited stakeholders’ engagement and discouraged project leads and staff while also limiting the upscaling and uptake of projects (P5 and P6 in Guadeloupe; P22 in New Caledonia; Supplementary Material 3). Another example is the lack of established monitoring techniques, which reduced both the potential effectiveness and the political and public support to a vegetation restoration project in Reunion Island (P13; Supplementary Material 3).

Impacts operated at two distinct levels (Figure 4C; Supplementary Material 4). At the project level, by constraining and delaying implementation, reducing potential effectiveness, hindering the monitoring of outcomes, increasing costs and discouraging the staff. And at territory level, firstly by affecting durably the capacities to implement EbA (e.g. through negative effects on collaboration and on political and public support); and secondly, by directly and negatively impacting the future uptake of EbA, due to project failure or the discouragement of project leads. These two levels of impacts are interrelated, as impacts at the project level (e.g. reduced potential effectiveness or increased costs) can generate impacts at the territory level (e.g. decreased EbA uptake).

### Temporalities of barriers

**Figure 6.**
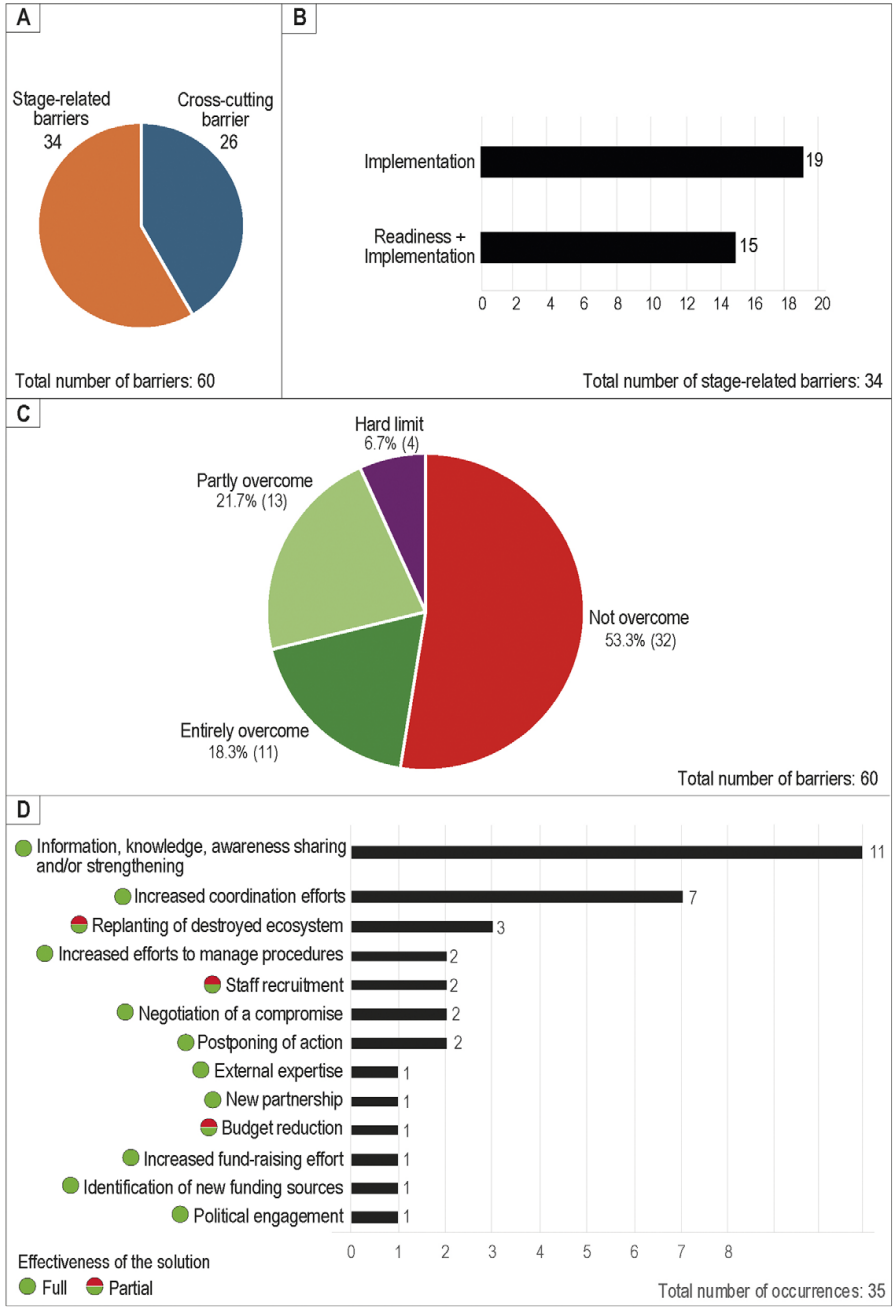
Temporalities of barriers. Most barriers are stage related (Panel A) and arise at the readiness and implementation stages of the adaptation process, with many barriers operating across these two stages (Panel B). The mutability of barriers is highlighted through the analysis of the frequency of not overcome, entirely overcome and partly overcome barriers, and identification of hard limits in the 24 EbA projects sample (Panel C). Panel D shows the 13 solutions that led to entirely or partly overcoming the barriers, their frequency of use and their effectiveness. Figure 6. long description.

First, the analysis of temporalities distinguishes between stage-related and cross-cutting barriers (Figures 6A). Thirty-four barriers out of 60 are stage related, that is, affect one or two stages of the EbA process, with 19 barriers affecting project implementation and 15 barriers affecting both readiness and implementation (Figure 6B; Supplementary Material 4). For example, difficulties in establishing compromises to manage conflicting interests (P12; Supplementary Material 3), or the lack of environmental sensitivity among stakeholders (P2, P9, P18, P21 and P22; Supplementary Material 3), negatively affected readiness and implementation. The 26 other barriers are cross-cutting, including, for example, difficulties in managing the cumbersome administrative and regulatory national and European procedures applicable to EbA funding and implementation, and the lack of awareness and knowledge on ecosystem functions. None of the identified barriers only affected the understanding/design stage, as barriers affecting this stage also negatively impacted readiness and implementation and were therefore cross-cutting.

Second, the analysis of the mutability of barriers reveals that 53.3% of barriers (32/60) were not overcome over the lifetime of the project, whereas 21.7% (13) were partly overcome, and 18.3% (11) entirely overcome (Figure 6C). The remaining barriers (6.7%; 4) constitute hard limits, including physical (limited site accessibility; limited available space to deploy the project), climatic (devastative flooding at the river mouth) and ecological (related to the lifecycle of species) limits.

Third, 13 different solutions were used to overcome barriers, some of which were implemented in several projects (35 occurrences; Figure 6D). The two most-used solutions were information, knowledge and awareness sharing or strengthening (11 occurrences representing 31.4% of implemented solutions) and increased coordination efforts (7 occurrences; 20%). Project leads reported that these two solutions were generally successful in overcoming barriers. Other solutions have a smaller number of occurrences (1 or 2) and mainly include (i) increased efforts to manage constraints (e.g. efforts to raise funds, manage procedures, use external expertise, recruit staff, develop partnerships, reduce budget, gain political support and negotiate a compromise with users), (ii) the repetition of the technical actions that had failed (e.g. replanting of mangrove propagules) and (iii) the postponing of actions that could not be deployed within the planned timeframe due to circumstantial factors, including the COVID-19 pandemic (all islands) and strikes (Guadeloupe). The less-effective solutions were the replanting of destroyed ecosystems where unfavourable hydrodynamic or climatic conditions occur, the recruitment of staff through subsidised jobs that did not meet EbA skill requirements and budget reduction negatively affecting the impact of the project.

## Discussion and conclusions: Lessons learnt for EbA research and practice

This study is the first to propose a structured analytical framework aimed at better understanding barriers to adaptation and to conduct a large-scale and grounded analysis (24 projects across five island territories) of barriers to EbA in tropical islands. The results generated, first, highlight the root barriers and their cascading effects on other barriers, as well as the genericity (to all island territories) or specificity (to a territory or region) of barriers; and second, demonstrate the value of integrating an analytical barrier-oriented framework applicable at project level into adaptation studies.

### Island- and adaptation-specific root barriers to EbA

In French overseas island territories, EbA faces major contextual barriers. The main one, faced by all projects, is the lack of awareness and knowledge about climate change risk and the specific requirements of EbA as an adaptation policy. It constitutes an island- and adaptation-specific root barrier, due to the backwardness of adaptation policies in these territories compared to mainland France. This barrier includes knowledge gaps on coastal dynamics, future climate risks, the protection service provided by ecosystems and the spatial–temporal conditions under which they can provide it (e.g. required spatial scale, and lead time for full effectiveness). Removing this adaptation-specific root barrier is crucial to overcoming other barriers. The first one is the limited adaptation-oriented institutional capacities, including the lack of identification of the institution(s) responsible for EbA, the lack of staff specifically dedicated to adaptation implementation and the lack of regulations and tools facilitating the setting up and implementation of EbA, observed in all territories. In French overseas departments (Martinique, Guadeloupe and Reunion Island) having the same administrative organisation and laws as mainland France, the challenges posed by this institutional gap are exacerbated by the complexity of the national and European administrative and regulatory procedures, and of associated funding rules, applicable to EbA. Yet, the institutional gap is most pronounced in French Polynesia, where EbA project leads highlighted the lack of designated institution in charge of EbA, which they attributed to the absence of a public agency responsible for coastal matters (e.g. a coastal observatory or conservatory, as exists in other territories). Addressing these institutional and knowledge gaps would in turn help address the lack of political support to EbA in the face of socioeconomic challenges that are locally perceived by elected representatives and practitioners as more urgent than adaptation, and which include employment, pollution management and water security (Terorotua et al., 2020). Another barrier that could be overcome by strengthening climate risk and EbA-related knowledge is the lack of technical and scientific expertise and resources to implement EbA, which was noted in all territories. This would also help leverage public support to and engagement with EbA, especially in urban and peri-urban areas where EbA remains misunderstood. Moreover, strengthening the understanding of EbA requirements would in turn make on-site knowledge a priority to support EbA and thereby support the generation of currently lacking baseline data. Another barrier that could be removed by strengthening climate risk and EbA-related knowledge relates to funding, including insufficient EbA-oriented funding and inappropriate funding tools constraining action and flexibility on the ground. These findings, which apply to all studied island territories, clarify what the lack of human, technical and scientific, and financial capacities of island countries and territories highlighted in the literature (e.g. Mycoo et al., 2022; Robinson, 2020) concretely mean on the ground. Beyond small island specificities, this shows the major role of policies and strategies, and legislation, as enabling factors for EbA (Pontee et al., 2016). Moreover, contrary to what is often claimed and in line with two recent studies (Osaka et al., 2021; Duvat et al., 2025), these findings emphasise that EbA is neither easy to implement nor inexpensive in tropical islands.

Furthermore, this study shows that these major barriers reduce EbA potential effectiveness while also constraining and delaying implementation, thereby generating not only immediate but also, and this has not been highlighted by previous studies, long-lasting negative effects on the desirability and success, and therefore uptake, of EbA. Barriers thus create a vicious circle effect that must be urgently addressed for EbA to remain desirable and promising in tropical island settings, where they have the potential to significantly reduce climate risk and be cost-effective (Fabian et al., 2014; Beck et al., 2018).

### Three ways forward to improve EbA practice in tropical islands

Three main lessons emerge from the analysis of the barriers to EbA in French tropical island territories, which are relevant to other small islands.

First, our findings call for prioritising the effective protection of still-healthy and functional ecosystems by reducing the human pressures that degrade them over prevailing restoration actions. In our sample, two projects (P9 and P17; Supplementary Materials 1 and 3) failed, highlighting the hard limits faced by coral restoration in highly degraded marine environments where the root causes of degradation are not addressed. This observation is not unique to island regions but critical to the success of EbA globally (e.g. Duarte et al., 2020; Seddon et al., 2020a, 2020b).

Second, among the 24 EbA projects analysed, few included monitoring and none used indicators measuring effectiveness. Yet, establishing shared monitoring protocols and putting them into practice is crucial to generate field evidence of the success of EbA, enhance the usefulness of experience sharing and thereby support EbA uptake and amplification (Narayan et al., 2016; Duvat et al., 2025). This means shifting from the ecological indicators currently in use (e.g. survival rate and growth of plant species) to geomorphological indicators measuring risk reduction. Examples of risk-relevant geomorphological indicators are the vertical accretion rate of the substrate in protected and restored mangrove areas, and the upward and seaward growth of beach crests and dunes where the coastal vegetation is effectively protected and managed. Such ground-based evidence would help remove several barriers to EbA, including the lack of political and population support, limited stakeholders’ engagement and limited EbA-oriented funding.

Third, communicating more broadly and effectively the benefits of EbA to the public and concerned stakeholders (Temmerman et al., 2013), for example, through participatory methods involving them in implementation and monitoring, would reduce conflicts, increase engagement, and support EbA upscaling and uptake.

### Integrating the assessment of barriers in adaptation studies

Because barriers are context specific and mutable, removing them first and foremost requires understanding them properly across the various stages of EbA projects and assessing the effectiveness of the solutions deployed to overcome them. This in turn calls for the systematic integration of a barrier-oriented assessment into EbA and other adaptation solution-oriented studies. The new four-step framework proposed in this article can serve as a starting point to fill this major research and practice gap. Because it allows for successive iterations over the lifetime of EbA projects, this framework can be used to assess the progress made in overcoming barriers. Furthermore, because it is not specifically designed for EbA but is generic in nature, it can also be used to monitor and evaluate other types of coastal adaptation measures, such as relocation or accommodation. This would make it possible to determine which barriers are specific to EbA and which ones are not.

## Supporting information

10.1017/cft.2026.10035.sm001 Duvat supplementary material 1 Duvat supplementary material

10.1017/cft.2026.10035.sm002 Duvat supplementary material 2 Duvat supplementary material

## Data Availability

All data generated or analysed during this study are included in this published article and its Supplementary Material files. Additional data on the adaptive performance of study EbA projects are provided in a previous article (Duvat et al., 2025). Non-academic deliverables are available from the author on reasonable request.

## Supplementary material

The supplementary material for this article can be found at http://doi.org/10.1017/cft.2026.10035.

## Inclusivity statement

This study was undertaken to meet the demand expressed by local to national actors involved in the design and implementation of coastal ecosystem-based adaptation projects (EbA) in French overseas island territories. Representatives of local and national governments, local associations, NGOs and private tourism companies leading, participating in and funding such projects provided grey literature and data that allowed the author to conduct this study. Eight of them participated in the steering committee that was created to discuss and validate the whole scientific approach. The analysis of EbA projects comprised two steps, including a first assessment which was shared with project leads, who commented and validated the results generated to then come up with a final assessment. The findings were shared with concerned stakeholders through 1- to 3-day on-site workshops including knowledge sharing sessions, on-site and indoor presentations by project leads and partners, detailed presentations of the results of the assessment by VKED, discussion sessions, and a final session dedicated to drawing up a roadmap for future activities. In addition, concerned stakeholders, including public and private actors, were trained in the use of the methodology, and transfer activities organised. Last, the results of the study were shared with the population through public conferences. In 2024, the assessment method was used by the French Ministry of Ecology to assess the projects submitted to the 2024–2029 call for projects “*Nature-based Solution to contain coastal erosion.*” The conclusions of this study are used by stakeholders to strengthen EbA capacities locally.

## Figure 1. Long description

Maps showing the location and nature of EbA projects deployed in French overseas tropical island territories, with the various technical interventions implemented, in Caribbean Islands (Guadeloupe and Martinique), Reunion Island (Indian Ocean) and the Pacific Ocean (New Caledonia and French Polynesian islands: Moorea, Tahiti and the Tuamotu atolls).

Navigate back to Figure 1..

## Figure 2. Long description

Panel A considers seven tropical ecosystems, including mangroves, coral reefs, swampy forests, inland forests, seagrasses, saltmarshes and beach-dune systems. The prevalent ecosystems targeted by the EbA measures are beach-dune systems (55.17%) and mangroves (20.68%). Panel B highlights the number of ecosystems targeted by eEbA-based interventions. It shows that most projects (79.2%) consider one single ecosystem. Panel C describes the types of technical interventions undertaken. It highlights the prevalence of actions combining improved ecosystem management and ecosystem restoration (54.2%) and ecosystem restoration alone (41.7%).

Navigate back to Figure 2..

## Figure 3. Long description

The analytical framework comprises four steps (boxes in the figure) representing the four interrelated dimensions that adaptation barrier studies should consider. The arrows show how the figure reads. This analytical framework proposes an iterative approach in which barrier analysis describes their nature (categories and sub-categories of barriers), origins (objective vs subjective character, level of occurrence, and (non)adaptation specificity), impacts on the adaptation process (type of impact generated and level of impact, namely project or territory level) and temporalities (stage of manifestation, mutability and solutions that allowed to overcome them). This framework allows us to describe barriers at various stages of an adaptation project and thereby monitor the capacity of project leads and partners to increase their adaptation potential.

Navigate back to Figure 3..

## Figure 4. Long description

Panel A is a graph showing the number (in orange) and frequency (in blue) of barriers for each of the six categories considered and the prevalence of barriers related to institutions, governance, politics, laws and regulations (category 1) and awareness, knowledge and technical resources (category 2). Panel B is a graph detailing by sub-category the results shown in Panel A. Panel C is a graph highlighting the 13 types of impacts of barriers on ecosystem-based adaptation projects, with reduced effectiveness and delay in implementation being the two predominant impacts.

Navigate back to Figure 4..

## Figure 5. Long description

Panel C is a graph emphasising that 36.7% of barriers to ecosystem-based adaptation projects only are adaptation-specific. The four adaptation-specific barriers are displayed in Panel D, with 59.5% of them related to the lack or weakness of ecosystem-based adaptation-oriented policy and tools.

Panel B is a graph highlighting the level of occurrence of barriers, especially their context-specificity.

Panel A is a graph showing the distribution of barriers per category according to their objective (80% of barriers) versus subjective nature.

Navigate back to Figure 5..

## Figure 6. Long description

These graphs show that barriers are both stage related and cross-cutting (Panel A), mostly related to the readiness and implementation stages of ecosystem-based adaptation projects (Panel B), difficult to overcome with 53.3% of them that were not overcome over the project lifetime and 6.7% constituting hard limits (Panel C). For those that were entirely or partly overcome, information, knowledge and awareness strengthening/sharing and increased coordination efforts were the most effective actions (Panel D).

Navigate back to Figure 6..
