# Supplementary material for: Assessing barriers to ecosystem-based adaptation: Application to tropical islands
Source: Camb Prism Coast Futur. 2026 Jun 3;4:e14. doi: 10.1017/cft.2026.10035 (PMC13276724; doi:10.1017/cft.2026.10035)
Supplement: Duvat supplementary material 1 — Duvat supplementary material [file S2754720526100353sup001.docx]

**Journal:** Cambridge Prisms Coastal Futures

**Supplementary Material to article “**Assessing barriers to Ecosystem-based Adaptation: application to tropical islands”

**Virginie. K.E. Duvat –** [virginie.duvat@univ-lr.fr](mailto:virginie.duvat@univ-lr.fr)

**Content of Supplementary files:**

SM1 – Description of Ecosystem-based Adaptation projects implemented in French Overseas Tropical Island Territories

SM2 – Main sections of the interview guide used to document EbA projects

SM3 – Description of barriers to Ecosystem-based Adaptation projects

SM4 – Database on barriers to Ecosystem-based Adaptation projects

**Supplementary Material 1** – Description of ecosystem-based Adaptation projects implemented in French Overseas Tropical Island Territories

This table details the main characteristics of study projects, derived from document analysis, interviews and fieldwork. The last column presents the performance of projects in meeting adaptation requirements.

| CARIBBEAN | | | | | | | |  |
| --- | --- | --- | --- | --- | --- | --- | --- | --- |
| **Project No.** | **Location (Territory + site)** | **Project name and location**  **Degree of technical mastering (experimental, mastered, transferred)** | **Date of implementation (and date of first action if any)** | **Main goal(s)** | **Description of technical actions** | **Project holder** | **Funding source (and total cost if known)** | **Adaptive performance (based on Duvat et al., 2025)** |
| P1 | Martinique  Fort-de-France | Z’Ab marina  Experimental | 2018-2026 | -Reduce wave height in the harbor basin by supporting the extension of the mangrove  -Demonstrating that Nature-based Solutions are compatible with economic activities | -Installation of a technical disposal encouraging vertical accretion in shallow waters through the increase of bottom roughness (bamboo fascines, wooden breakers, biodegradable nets), aimed at enabling mangrove extension over a 250m^2^ area  -Planting of mangrove if necessary | Community of municipalities (CACEM) | EU Life ARTISAN project (480,000€) | 60.00% |
| P2 | Martinique  Sainte-Anne | Boucaniers Village  Experimental | 2012-Present | -Reduce current to future coastal erosion  -Prevent marine flooding | -Removal of groynes  -Installation of shore-parallel sandbags to stabilize the upper beach  -Restoration of indigenous coastal vegetation | Tourism company | Club Med (not estimated) | 44.30% |
| P3 | Martinique  Le Lamentin | RÉCIPROCITÉ Experimental | 2016-2025 (first actions in 2013) | -Restoring back mangrove functionalities, especially flood control  -Promoting sustainable relationships between the environment, people and local economic activities | -Inventory of biodiversity  -Awareness raising among schoolchildren and educational initiatives  -Removal of exotic invasive species  -Reforestation of the back mangrove (10 hectares)  -Promoting eco-responsible practices among economic actors  -Enhancement and renaturation of an islet through cultural and educational actions  -Removal of end-of-life boats from the bay and promotion of sustainable mooring management  -Enhancement of the Longvilliers River (promoting sustainable management from the watershed to the sea) | Municipality of Le Lamentin | Life4BEST, OFB (French Office for Biodiversity), AFD (French Development Agency), municipality of Le Lamentin (1M€) | 71.10% |
| P4 | Guadeloupe  Cluny Beach, Anse Maurice Beach, Anse à la Gourde Beach, Salines Beach  Multi-site | Carib Coast  Transferred | 2019-2023 (first action in 2007) | -Reduce current to future coastal erosion  -Generate and disseminate knowledge on sustainable beach management  -Create a network of experts on coastal risks and adaptation to climate change | -Production of a best practice guide on beach management  -Beach vegetation restoration (enclosures + planting)  -Awareness raising and knowledge sharing | National Forestry Office (ONF) | EU FEDER, National Forestry Office (ONF) (322,135€) | 63.90% |
| P5 | Guadeloupe  Port-Louis | Renaturation of North Port-Louis coastal area  Transferred | 2020-2022 (first action in 2000) | -Reduce coastal erosion  -Reconnect the mangrove to the sea  - Support the emergence of a nature-centric vision of the coastal zone | -Hydrological and geomorphic studies  -Ecological inventories  -Coastal road closure and reorganization of vehicle parking and travel in favor of soft mobility  - Beach vegetation restoration (enclosures + planting) | National Forestry Office (ONF) | State, National Forestry Office (ONF) (264,400€) | 65.00% |
| P6 | Guadeloupe  La Saline Beach,  Le Gosier | Experimental approach to soft coastline management Experimental | 2012- Present (first action in 2007) | -Reduce barrier beach erosion to protect a biodiversity priority area (swamp)  -Demonstrate that Sargassum deposits are not necessarily and only detrimental  -Promote soft coastline management | -Multidecadal shoreline change study and monitoring  -Coastal road closure and reorganization of vehicle parking and travel in favor of soft mobility  -Maintenance of Sargassum leaves on the beach and promotion of sustainable beach management practices and control of beach uses with the aim of supporting the re-establishment and growth of the indigenous vegetation | Le Gosier municipality, French Coastal Conservatory (CDL) | Le Gosier municipality, French Coastal Conservatory (CDL) (not estimated) | 52.20% |
| P7 | Guadeloupe  Sainte-Anne | Reducing coastal risks at Caravelle Village  Experimental | 2014-Present | -Reduce current to future coastal erosion  -Maintain beach attractivity to support beach tourism | -Shoreline and beach change study  -Removal of a pontoon obstructing alongshore sediment transfer | Tourism company | Club Med, not estimated | 39.40% |
| P8 | Guadeloupe  Jarry  Multi-site | Ja-riv  Experimental | 2016-Present | -Reduce current to future coastal flooding  -Preserve and restore biodiversity | -Diagnostic of illegal occupations of the Public Maritime Domain by private companies  -Initiation and follow-up of land release by private companies  -Restoration of released land plots (decontamination, decompaction, mangrove restoration)  -Raising awareness about the ecological functions of swamp areas (development of a coastal path and knowledge sharing) | French Coastal Conservatory (CDL) | BRGM (Bureau of Geological and Mining Research), DEAL of Guadeloupe (State environmental division), EU FEDER, Water Office (ODE), French Office for Biodiversity (OFB), TotalEnergies Foundation (9,250,000€) | 59.40% |
| P9 | Guadeloupe  Pointe-à-Pitre (Petit Cul-de-Sac Marin) and Folle Anse harbour (Marie-Galante Island)  Multi-site | Adapt’Island  Experimental | 2019-2024 (first action in 2013) | -Promote an innovative ecosystem-based climate adaptation strategy and disseminate innovative ecosystem restoration techniques  -Reduce flooding  -Restore and protect marine and coastal ecosystems  -Restore ecological continuity between marine and coastal ecosystems  -Involve local stakeholders and the civil society and raise awareness about ecosystem functions | -Establishment of a technical and scientific strategy for the Grand Port of Guadeloupe  -Mangrove restoration (80,000m^2^) through the removal of exotic invasive species, soil decompaction and decontamination  -Establishment of eco-friendly anchoring in seagrass area  -Coral transplantation to support coral reef restoration in Petit Cul-de-Sac Marin | Grand Port of Guadeloupe (GPMG) | EU Life, Grand Port of Guadeloupe (4,726,970€) | 62.20% |
| P10 | Guadeloupe  Saint-François | Plant’Aksyon  Mastered | 2021-2022 (first action in 2018) | -Reduce current to future coastal erosion  -Awareness raising  -Protect biodiversity (flora and turtles) | -Construction of plant nurseries and training of schoolchildren to take cuttings  -Restoration of the indigenous coastal vegetation (enclosures and planting) | Saint-François municipality, 3 schools involved in Educative Marine and Terrestrial Areas | EU Life4BEST (35,775€) | 65.00% |

| INDIAN OCEAN | | | | | | | |  |
| --- | --- | --- | --- | --- | --- | --- | --- | --- |
| **Project No.** | **Location (Territory + site)** | **Project name and location**  **Degree of technical mastering (experimental, mastered, transferred)** | **Date of implementation (and date of first action if any)** | **Main goal(s)** | **Description of actions** | **Project holder** | **Funding source (and total coast if known)** | **Adaptive performance (based on Duvat et al., 2025)** |
| P11 | Reunion Island  Saint-Paul | Rehabilitation of Cambaie Beach  Experimental | 2019-2023 | -Reduce current coastal erosion  -Restore turtle nesting sites | - Removal of exotic tree species  -Beach reprofiling  -Planting of forest species (300m)  -Awareness raising | Municipality of Saint-Paul and the National Forestry Office | State and municipality (125,000€) | 66.70% |
| P12 | Reunion Island  Saint-Paul | Enhancement and restoration of the wooded coastal fringe of l'Hermitage-les-Bains  Experimental | 2017-2023 (first actions in 2003) | -Reduce current coastal erosion  -Strengthen biodiversity  -Raise public awareness on biodiversity preservation  - Improving the quality of life for residents and tourists  -Encourage soft mobility practices | -Removal of exotic tree species  -Beach nourishment and reprofiling  -Forest species planting (2,200m) on the upper beach and back beach  -Awareness raising  -Control of uses, including user path management | Saint-Paul municipality | EU (FEDER), municipality, community of municipalities (3,630,802€) | 55.00% |
| P13 | Reunion Island  Étang-Salé | Rehabilitation of a turtle nesting site at Gouffre de l’Étang-Salé  Experimental | 2010-2013 (first actions in 2007) | -Reduce current to future coastal erosion  -Restore turtle nesting sites | -Removal of exotic invasive species  -Beach reprofiling  -Restoration of the coastal indigenous forest (9.25 hectares, 250m)  -Promotion of sustainable beach use | National Forestry Office | EU (FEDER), National Forestry Office (300,000€) | 58.30% |
| P14 | Reunion Island  West coast  Multi-site | Rehabilitation of sea turtle nesting sites on the West Coast  Transferred | 2017-2022 (first actions in the 1990s) | -Reduce current to future coastal erosion  -Restore turtle nesting sites  -Raise public awareness about proper beach management  -Federate stakeholders concerned by beach preservation  -Improving the living environment | -Removal of exotic plant species  -Beach vegetation planting and restoration in several sediment cells  -Awareness raising  -Training and capacity building  -Knowledge strengthening | Center for the Study and Discovery of Marine Turtles (CEDTM) | EU (FEDER), Region, State (746,000€) | 77.20% |
| P15 | Reunion Island  Saint-Paul | Sediment management et Saint-Gilles Beach | 2017-Present | -Reduce current coastal erosion  -Manage sediment accumulation in harbor basin | -Beach nourishment along 280 m of shoreline  -Sediment dredging from harbour basins | Community of Municipalities of the Western Coast | Community of Municipalities of the Western Coast (total cost unknown) | 57.80% |

| PACIFIC OCEAN | | | | | | | |  |
| --- | --- | --- | --- | --- | --- | --- | --- | --- |
| **Project No.** | **Location (Territory + site)** | **Project name**  **Degree of technical mastering (experimental, mastered, transferred)** | **Date of implementation (and date of first action if any)** | **Main goal(s)** | **Description of actions** | **Project holder** | **Funding source (and total cost if known)** | **Adaptive performance (based on Duvat et al., 2025)** |
| P16 | French Polynesia  Moorea | RESCCUE  Experimental | 2015-2020 | -Reduce current to future coastal erosion  -Support recreational activities  -Raise awareness about coastal erosion among beach users and implement participatory beach management | -Creation of a rock-made sediment trap in the intertidal area  -Beach nourishment (350 m of shoreline, 5300m^3^)  -Restoration of indigenous coastal vegetation | Tourism Department of the Government of French Polynesia, National Biodiversity Office | Tourism Department of the Government of French Polynesia, National Biodiversity Office (390 | 67.20% |
| P17 | French Polynesia  Moorea & Tahiti, Society Islands; Ahe & Tikehau, Tuamotu-Gambier Islands  Multi-site | Coral reef restoration by Coral Gardeners Transferred | 2017-Present | -Preserve the reef ecosystem  -Preserve the ecosystem services provided by coral reefs, including the coastal protection service  -Improve people's standard of living by creating jobs and preserving marine resources (e.g. fish stocks) | -Large-scale coral transplantation (1 million coral cuttings by 2025)  -Awareness raising | Coral Gardeners Association | Donations, partnership, public funding, sponsoring (approx. 1.2M€/year) | 58.30% |
| P18 | French Polynesia  Hao Atoll, Tuamotu-Gambier Islands | Restoration of the ocean beach crest's native vegetation  Experimental | 2022-2023 | -Reduce coastal erosion and marine flooding  -Preserve biodiversity and remove invasive exotic species  -Promote local development through job creation and equipment purchases | -Coastal vegetation restoration through planting: 1650m, 3 spots  -Awareness raising  -Youth training | Municipality of Hao | National Office for Biodiversity + Municipality of Hao (179,527€) | 54.40% |
| P19 | French Polynesia  Paea, Tahiti, Society Islands | Restoration of the native coastal vegetation, including sub-mangrove, coastal forest and salt meadow  Experimental | 2021-2023 | -Reduce current to future coastal erosion  -Preserve biodiversity  -Propose a standardized restoration protocol for degraded coastal areas | -Vegetation planting (5,000 m^2^)  -Awareness raising | Te Ora Naho (Fédération des Associations de Protection de l'Environnement), French Polynesian Government Research Delegation | EU BEST2.0+ (and volunteering and mutual aid) (60,000€) | 53.90% |
| P20 | French Polynesia  Anaa Atoll, Tuamotu-Gambier Islands | Restoration of the Tukuhora Priority Conservation Area Experimental | 2021-2024 | -Reduce cyclone-induced marine flooding  -Protect the primary forest from human pressures (airport, village extension).  -Strengthen the ecosystem services associated with this vegetation formation: medicinal, socio-economic and cultural | -Forest vegetation planting (400 m^2^)  -Sustainable forest management: fire prevention, removal of waste from the former landfill (3 m^3^), reduction of vegetation cuttings within the airport perimeter  -Awareness raising  -Reintroduction of the endemic pupu rega snail | Local association Pu tahi haga no Ganaa | National Office for Biodiversity + International Union for Nature Conservation (IUNC) (73,300€) | 43.90% |
| P21 | New Caledonia  Nouméa, Grande Terre | Mangrove and beach vegetation restoration at Tina Golf by SOS Mangroves  Experimental | 2017-2023 | -Reduce current to future coastal erosion  -Preserve mangroves  -Support social reintegration  -Raise awareness about mangrove related ecosystem services | -Planting of 8 hectares of mangroves and 150m of beach vegetation  -Reconnection of the mangrove to the sea  -Networking to promote capacity building  -Reintegration projects | SOS Mangroves association | Multiple funding sources, including mutual aid and volunteering (not estimated) | 47.20% |
| P22 | New Caledonia  Bourail, Grande Terre  Multi-site | Coastal vegetation restoration by the Bwärä Tortues Marines association  Transferred | 2006-Present | -Reduce current to future coastal erosion  -Protect turtle nesting sites  -Reconnect marine and coastal ecosystems | -Coastal vegetation planting (13 hectares)  -Removal of car access to beaches  -Awareness raising  -Networking to promote capacity building | Bwärä Tortues Marines association (Bourail, Grande Terre) | Multiple funding sources, including mutual aid (not estimated) | 63.30% |
| P23 | New Caledonia  Touho, Grande Terre | Mangrove restoration by the Hô-üt association Transferred | 2022 (previous actions started in 2013) | -Reduce current to future coastal erosion and marine flooding  -Raise awareness about mangrove related ecosystem services | -Mangrove planting (150m and 2,500 m^2^)  -Awareness raising | Hô-üt association | International Union for Nature Conservation (IUNC), Pacific Regional Environment Programme (SPREP), volunteering (8,000€) | 64.40% |
| P24 | New Caledonia  Poindimié, Grande Terre | Mangrove and coastal vegetation restoration by the Popwadene association  Experimental + Transferred | 2020-2023 (first similar actions started in the 1980s) | -Reduce current to future coastal erosion  -Preserve a major cultural, feeder and recreational site | -Mangrove planting (9,000m^2^)  -Coastal forest restoration (300m)  - Awareness raising | Saint-Denis tribe  Popwadene association | EU BEST 2.0+ (51,391€) | 73.90% |

**Supplementary Material 2** – Main sections of the interview guide used to document Ecosystem-based Adaptation projects.

This table summarises the main sections (left column) and sub-sections (right column) of the interview guide used to document coastal Ecosystem-based Adaptation projects. The semi-structured interviews were conducted with project leads and their partners (3 to 5 interviews per project).

| 1. Project’s location | Overseas territory |
| --- | --- |
|  | Island |
|  | Municipality/community |
|  | Site |
| 2. Origin of the project | Reasons for/context of design and implementation, initiator |
|  | Previous actions implemented at the same site to address coastal risks |
| 3. Context | Policies, regulations and tools applying in relation with the site and the project (local and national, if relevant) |
|  | Land tenure at project location and nearby |
|  | Technical readiness at the territory’s scale |
|  | Cognitive context at the territory’s scale |
| 4. Description of project | Project lead and partners (internal and external) and their respective roles in project implementation; collaborations and networking |
|  | Main and secondary goal(s) of the project, including the coastal risks targeted |
|  | Understanding of risk and how the project can reduce it by project holder and partners |
|  | Technical actions, and their spatial and temporal scales |
|  | Non-technical actions, and their spatial and temporal scales |
|  | Project’s governance and related modalities |
|  | Previous, emerging or expected conflicts/conflictual issues |
|  | Social acceptability, public society’s involvement in the project, and empowerment |
|  | Project’s cost, funding source(s), economic evaluation |
|  | Human and technical capacities of project holder and partners |
|  | Support provided by studies and monitoring, and project’s final evaluation |
|  | Strategic and adaptive dimension |
|  | Co-benefits and beneficiaries; disbenefits and losers |
|  | Lock-ins and maladaptive effects |
|  | Levers and barriers to implementation and success |
|  | Solutions to barriers and outcomes |

**Supplementary Material 3 – Description of barriers to Ecosystem-based Adaptation projects**

The barriers were identified based upon semi-structured interviews and workshop discussions with Ecosystem-based Adaptation projects leads and their partners. In this table, the 60 inventoried barriers are categorised by type (e.g. 1) and sub-type (e.g. 1.1). The same numbering system is used in Supplementary Material 4. The projects facing these barriers are indicated into brackets (e.g. P1). The numbering and description of projects is presented in detail in Supplementary Material 1. The numbering, name and territory of deployment of projects is summarised below this table.

| BARRIERS |
| --- |
| 1. Institutions, governance, politics, laws and regulations, including land tenure |
| Institutions and regulations |
| 1.1 Failure in contracting with a public research institution (University of Antilles and Guyane) for expertise and monitoring due to the cumbersome nature of the required procedure (P1) |
| 1.2 Cumbersome national administrative and regulatory procedures, in particular relating to public procurement (P1, P9, P15), technical interventions on the public maritime domain (P3) and on coastal conservatory land (failure to set up monitoring on Cluny Beach) (P4), technical interventions involving the handling of protected species (corals, P9; coastal vegetation, P11, P13, P14), the dismantling of engineered structures (P7), the environmental impact assessment procedure required for projects with a budget > €160,000 (P1), the validation of the project (P12) |
| 1.3 Cumbersome administrative procedures (including reporting) relating to the European LIFE (P9) and LIFE4BEST (P10) projects; difficulties in meeting European projects standard (P9, P10) |
| 1.4 Complexity of multi-partner projects (P2, P4, P5) |
| 1.5 Lack of regulatory tools to support EbA implementation (P20) |
| 1.6 Lack of implementation of existing regulatory tools related to coastal development (P8) and waste and wastewater management (P3, P6, P8, P9, P17, P19, P21, P22) |
| 1.7 Absence of public institutions (e.g., Coastal Conservatory, National Forestry Office) to carry out and support large-scale EbA projects (French Polynesia; P16, P18, P19) |
| 1.8 Limited human capacity of public institutions involved in EbA to ensure maintenance and monitoring (P8, P16, P20) |
| Land tenure |
| 1.9 Complexity of multi-stakeholder land management (P1, P9, P11, P13, P20, P21) |
| Governance and politics |
| 1.10 Lack of clarity on responsibilities, notably about interventions on the public maritime domain (municipality vs. tourism company; P7) |
| 1.11 Lack of public (P4, P13) or political (P4, P20) support due to a lack of understanding of the causes of coastal erosion and of how the project could reduce it |
| 1.12 Conflict between the project leader and the municipality (P20) |
| 1.13 Difficulties in involving stakeholders: refusal of some public institutions to engage (community water and sanitation authority; P3), difficulty in involving all levels of decision-making and action (P6), difficulty in fully involving stakeholders ("timid involvement"; P8), a lack of interest and commitment (town hall and province) and conflicts (between SOS Mangroves association and the golf club; P21) |
| 1.14 Conflict between the private project lead (tourism company) and the municipality related to the modalities of intervention on the public maritime domain, funding, and the solution to be promoted (P7) |
| 1.15 High turnover in the municipality and companies in charge of setting up and implementing the project (P12) |
| 1.16 Difficulties in establishing compromises on sites involving multiple and competing interests (P12) |
| 1.17 Divergence in coastal management practices of public actors (National Forestry Office, Coastal Conservatory, municipalities) (P4, P6, P12) |
| 1.18 Marginalization of the project team within the structure (EbA are not its core business; P9) |
| 1.19 Lack of lead to manage the project (P18) |
| 1.20 Prioritisation of short-term issues (protection of human assets) over long-term issues (reconstitution of a buffer zone) (P19) |
| 1.21 Limited support and implication of the population (P4, P5, P20, P21) |
| 2. Awareness, knowledge, technical resources |
| Awareness |
| 2.1 Lack of awareness and knowledge about the specific requirements of EbA, including the spatial scale (multi-ecosystems, watershed scale) and timescales (lead time until full effectiveness, duration of benefits) of action; need for risk assessment, and for monitoring (assessing effectiveness) and maintenance (all projects) |
| 2.2 Lack of awareness of local institutions about the role of coastal vegetation in beach stabilisation ("cleaning") in Guadeloupe (P5, P6) and New Caledonia (P22) |
| 2.3 Lack of awareness and recognition of the protection provided by mangroves in urban and industrial areas (P3, P21) |
| Knowledge |
| 2.4 Uncertainties about the technical effectiveness of the project due to its innovative character (P1, P6) |
| 2.5 Lack of knowledge on some ecosystems (back mangrove; P3; and swamp forest; P8, P9) |
| 2.6 Lack of knowledge on invasive plant species, leading to the recommendation or planting of some of these species (P10, P12, P13) |
| 2.7 Lack of geomorphological (all projects) baseline to guide action |
| Technical resources |
| 2.8 Lack of technical expertise of the project lead (P7, P9, P12, P16, P20) |
| 2.9 Lack of technical expertise locally: creation of a neo-mangrove (P1); maintenance of Sargassum leaves on the beach surface to reduce erosion (P6); dredging, sediment transfer and beach recharge (P15); production of native plant species and reintroduction of these species into the environment (P16, P19); restoration of primary forest (P20); soil decontamination in industrial zones and bioremediation (P8, P9) |
| 2.10 Lack of established monitoring techniques (P13) |
| 2.11 Lack of technical skills to apply to calls for projects and to produce deliverables (P18, P20) |
| 2.12 Lack of equipment, including heavy plant machinery to conduct works (P15); lack of water storage facilities to water young plants (P11, P13, P16, P18, P20); lack of small equipment (e.g. pots) for mangrove plantation (P21) |
| 3. Financial and economic |
| Financial |
| 3.1 Cost of implementation significantly higher than allocated budget (P1) |
| 3.2 Financial rules of public institutions preventing operating expenses (municipalities; P3) and the recruitment of staff (Coastal Conservatory; P8) |
| 3.3 Limited budget preventing the recruitment of skilled staff (P14, P18, P19, P20, P21) |
| 3.4 Complexity of multi-partner funding (P2) |
| 3.5 Difficulties in identifying sources of funding and in raising funds (especially P12, P14, P16, P19, P20, P21, P23) |
| 3.6 Lack of flexibility of the rules related to the provision of services (limitation to 35% of the total budget) in European LIFE projects (P9) |
| 3.7 Inappropriateness of financial rules applying to European projects for Pacific territories (e.g. official invoices imposed vs. mutual aid system; P19) |
| 3.8 Inappropriateness of multi-year funding not providing the long-term human and technical resources required for EbA (P4, P5, P8, P9, P11, P13, P14, P16, P19) |
| 3.9 Insufficient EbA-oriented funding (P11, P12, P13, P14, 19) |
| Economic |
| 3.10 Prioritisation of the economy over the environment (P8, P9, P20, P21, P22) |
| 4. Social, cultural, psychological |
| 4.1 Rejection of EbA by the population, e.g. through vandalism (destruction of the protected enclosures of the restored upper beach plots) and theft of materials due to the strong opposition from users to the parking constraints imposed as part of the project (e.g. road access closure or reduction of parking facilities; P4, P5, P12); vandalism due to jealousy (recruitment on subsidised contracts; P20); theft of mangrove planting material (P21); illegal sand extraction from nourished beach (P16) |
| 4.2 Refusal of residents to accommodate monitoring activities on their property (P4) |
| 4.3 Unpopularity of technical measures: maintenance of Sargassum leaves on the beaches (P6), reintroduction of native species (P14), sand mining from the lagoon for beach nourishment (P16) |
| 4.4 Strong sense of place (population attachment to invasive plant species providing shade and being part of the landscape; P12) |
| 4.5 Lack of environmental sensitivity and connexion to the environment among the population and economic actors (P2, P9, P18, P21, P22) |
| 4.6 Island culture in favour of the "cleaning" (removal) of the coastal vegetation in Guadeloupe (P5, P6) and New Caledonia (P22) |
| 5. Physical, climatic, ecological |
| Physical |
| 5.1 Limited accessibility of some beach sites severely affected by invasive alien species (P14) |
| 5.2 High human (including urban) pressure exerted on vegetated (P5, P12, P16, P21) and coral (P17) restoration areas |
| 5.3 Physical limits to restoration (airstrip; P20) |
| Climatic |
| 5.4 Flood damage caused to mangrove restoration area (P23, P24) |
| 5.5 Intense rainfall destroying seedlings (P16; P19) |
| 5.6 Marked drought causing the failure of vegetation restoration (P13; P16; P20) |
| Ecological |
| 5.7 Timing constraints imposed by the biological cycle of selected coral species (P9) |
| 5.8 Predation (crabs) and algae deposits destroying young mangrove trees (P21) |
| 5.9 Highly degraded ecological conditions (P9, P17) causing the failure of reef restoration |
| 6. Circumstantial |
| 6.1 COVID-19 pandemic (P2, P10, P12) |
| 6.2 Strikes (Guadeloupe; P10) |

**Name and location of study NbS:**

***1. Caribbean region (P1-P10)***

P1: Z'Ab, Fort-de-France, Martinique

P2: Actions to combat coastal risks, Boucaniers Village, Martinique

P3: Reciprocity, Le Lamentin, Martinique

P4: CaribCoast Project, Guadeloupe

P5: Port-Louis North Coastal Renaturation Project, Guadeloupe

P6: Experimental approach to flexible coastline management, La Saline beach, Le Gosier, Guadeloupe

P7: Actions to combat erosion, Caravelle Village, Guadeloupe

P8: Ja-riv, Guadeloupe

P9: LIFE Adapt'Island, Guadeloupe

P10: Plant'Aksyon, Guadeloupe

***2. Reunion Island, Indian Ocean (P11-P15)***

P11: Rehabilitation of Cambaie Beach, Saint-Paul

P12: Enhancement and restoration of the wooded coastal fringe of Hermitage-les-Bains, Saint-Paul

P13: Ecological rehabilitation of a sea turtle nesting site, Étang-Salé, Le Gouffre

P14: Rehabilitation of sea turtle nesting sites, west coast

P15: Sediment management, Saint-Gilles-les-Bains

***3. Pacific Ocean (P16-P24)***

P16: RESCCUE, Tahiamanu Beach, Moorea, French Polynesia

P17: Coral Reef Restoration by Coral Gardeners, Moorea, Tahiti, Ahé, Tikehau, French Polynesia

P18: Restoration of the native coastal vegetation of the ocean beach, Hao Atoll, Tuamotu, French Polynesia

P19: Restoration of the native coastal vegetation, Paea, Tahiti, French Polynesia

P20: Restoration of the Tukuhora Priority Conservation Area, Anaa Atoll, Tuamotu, French Polynesia

P21: Tina Golf Mangrove Restoration, Nouméa, New Caledonia

P22: Revegetation of the coastline, Bourail, New Caledonia

P23: Mangrove restoration, Touho, New Caledonia

P24: My Heart is in the Mangrove: mangrove and coastal vegetation restoration, Saint-Denis tribe, Poindimié, New Caledonia

**Supplementary Material 4** – **Database on barriers to Ecosystem-based Adaptation projects**

See attached Excel database.

Supplementary Material 4 should be read along with Supplementary Material 3, as the designation of barriers in the tabs *origins, impacts,* and *temporalities* of the Excel database (Supplementary Material 4) is identical to their designation in Supplementary Material 3. This Excel database contains the semi-quantitative analysis.
