# Supplementary material for: Assessing barriers to ecosystem-based adaptation: Application to tropical islands
Source: Camb Prism Coast Futur. 2026 Jun 3;4:e14. doi: 10.1017/cft.2026.10035 (PMC13276724; doi:10.1017/cft.2026.10035)
Supplement: Peer review history

# Reviewer report: Review: Assessing barriers to ecosystem-based adaptation: Application to tropical islands — R0/PR2

**DOI:** 10.1017/cft.2026.10035.pr2

This paper presents the negative unintended effects of coastal Nature-based Adaptation Solutions that are often not discussed. While it has the potential to contribute to a broader understanding of coastal NbS, significant improvements are necessary. Firstly, I believe that the ‘case study’ format may not be the most suitable for this paper. Secondly, a methodology section is essential for clarity and rigor. Please refer to my detailed comments below:

1. There are various interpretations of what constitutes NbS. For instance, “greening the grey” is often regarded as an NbS, yet it incorporates engineering elements. Therefore, it would be helpful to clarify what you mean by NbS in this paper. This is very much needed in order to contextualise the results.

2. Lines 77-86: While I understand and agree with your point, there has been significant debate about the necessity of this new term. Some argue it may simply be a case of ‘mutton dressed as lamb’, which raises concerns about potential greenwashing (e.g. https://doi.org/10.1111/1365-2664.70130 and https://doi.org/10.1016/j.ufug.2021.127345). As you noted, many of the examples provided primarily pertain to habitat restoration, a concept that is not new. I believe it’s important to briefly acknowledge these differing perspectives either here or later in the discussion.

3. Lines 97-99: I would rephrase to say that they ‘often’ are inexpensive. If you consider beach nourishment and managed realignment as NbS (depending on your answer to my first comment), these can be huge multimillion projects.

4. Lines 126-134: The current text lacks sufficient detail about your methods. Instead of relying solely on the published methodology, I recommend adding a dedicated methods section to this article. Please specify which islands you studied, what data you collected, how you gathered it, and how you analysed the data. Without this information, the subsequent sections lack context, and readers shouldn’t be expected to refer to another paper for clarity on your work.

5. Line 181: Do the numbers in the brackets refer to projects or interviewees? Please clarify. A list of projects or interviewees would be helpful in the main text (ideally) or at least in the supplementary materials.

6. Lines 275-292: This paragraph addresses the challenges in implementing coastal Nature-based Adaptation Solutions rather than their negative unintended effects. Later in the conclusions, Lines 453-458, you explain quite well why these can be perceived as negative effects. However, as they are presented now in section 2, it does not come across that way.

7. Line 446-448: This should be mentioned at the beginning of the results: only 13 cases had negative effects, and thus only these 13 cases are presented.

8. I am not convinced about your reasoning for applying Rahman et al. (2023) categorisation of barriers to classify consequences. Additionally, you refer to them as drivers instead of barriers, which needs further explanation and justification. I believe using the term ‘driver’ makes more sense in this context, but it is important to clarify this and remain consistent with one term. The addition of methodology section could help you with this.

9. It is unavoidable to have some repetitions in the conclusion, but I would recommend synthesising Lines 466-472 and have some more general conclusions.

# Reviewer report: Review: Assessing barriers to ecosystem-based adaptation: Application to tropical islands — R0/PR3

**DOI:** 10.1017/cft.2026.10035.pr3

This manuscript examines a number of NbS projects in French Polynesia with the purpose of highlighting unintended negative outcomes of the projects and exploring reasons for these outcomes. Data that supports the study is contained in supplementary information and largely appears to be qualitative information. The data is not well structured or presented, so that much of the article reads as a string of anecdotal information, which are tightly packed and the major issues are not highlighted well. Is there any empirical evidence of the negative consequences, or is it only qualitative? Ultimately, the article identifies the same range of constraints/barriers to effective implementation as found in most coastal (and environmental) management projects. I was unable to decipher whether there was any unique aspects of the NbS as applied in these atoll examples that were specifically relevant to atolls, or indigenous communities or climate change. Ultimately, I could find no new findings in this article that justify its publication and recommend major revisions.

I have significant interest in this topic and was hoping to learn much from its contents. However, I was disappointed. The manuscript is not well-structured and does not seem to make novel or compelling arguments. I would recommend restructuring the manuscript with a focus on: outlining the methodology adopted (currently absent); introducing the ‘data’ in a more robust/systematic manner; and identifying novel outcomes of the study.

Furthermore, the title of the manuscript suggests it focusses on the negative effects of NbS. However, the general impression after reading the manuscript is that its emphasis is more on the barriers to effective NbS implementation. The negative consequences are briefly overviewed through anecdotal information, but manuscript does not provide a compelling stocktake of these negative outcomes supported by evidence.

As part of the revision I would urge the authors to undertake a hard edit of the written language. There are multiple compound sentences that are impenetrable. For example, Lines 77-87 is one sentence that is extremely dense, contains multiple ideas, and should be broken into several parts. Otherwise, the reader is lost from the outset. Lines 180-189 is another giant sentence that needs to be separated.

# Editor report: Recommendation: Assessing barriers to ecosystem-based adaptation: Application to tropical islands — R0/PR4

**DOI:** 10.1017/cft.2026.10035.pr4

**Affiliations:** Institute for Coastal and Marine Research, Nelson Mandela University, South Africa

Dear authors - Both reviewers provide useful inputs to improve the manuscript including the addition of a methods section, clarification on what is meant by NbS, and restructuring of the manuscript including the identification of the novel outcomes of the study. I agree with the comment that the emphasis should be barriers to effective NbS implementation as the negative outcomes are anecdotal and not supported by evidence. We look forward to a revised contribution.

# Editor report: Decision: Assessing barriers to ecosystem-based adaptation: Application to tropical islands — R0/PR5

**DOI:** 10.1017/cft.2026.10035.pr5

**Affiliations:** Geography, Cambridge University, United Kingdom of Great Britain and Northern Ireland

No accompanying comment.

# Reviewer report: Review: Assessing barriers to ecosystem-based adaptation: Application to tropical islands — R1/PR7

**DOI:** 10.1017/cft.2026.10035.pr7

The previous version of this paper has been completely revised, and this is essentially a resubmission. Although detailed responses to my earlier comments have not been provided, I can see that some have been addressed, while others remain pertinent in this version. Please refer to my detailed comments below:

1. Introduction: Currently, the text immediately dives into categorising barriers. I believe it would be helpful to include a few introductory sentences that emphasise the importance of focusing on barriers to Ecosystem-based Adaptation, in particular in coastal and island contexts. Perhaps some of the content from Lines 111-120, particularly the first sentence, could be moved up to provide more context.

2. Lines 84-86: Could you elaborate on what root barriers and proximate barriers are, beyond the examples provided in the brackets? Additionally, why is this classification significant?

3. Figure 1: Great figure! Although the number of icons exceeds the number of projects. I think it would be good to clarify that they indicate the interventions and not the specific projects themselves, as I assume a project might have included several interventions, if I have understood this correctly.

4. Lines 167-168: Was this done the same way on all the islands? Particularly thinking of interviews and workshops.

5. Results: You mention the projects in the brackets, but without an overview list of all the projects, it does not add much. As already mentioned in the earlier version I reviewed, please include a list of projects, at least in the supplementary materials. This would also help in interpreting Figure 1.

6. Line 349: Did any of the interviews elaborate on what they mean by ‘reduced effectiveness’? How was the effectiveness of EbA assessed?

7. Lines 436-439: These differences between islands are quite interesting. Did you observe any other differences, and how would you explain them?

# Reviewer report: Review: Assessing barriers to ecosystem-based adaptation: Application to tropical islands — R1/PR8

**DOI:** 10.1017/cft.2026.10035.pr8

This article provides an overview of barriers to ecosystem-based adaptation EbA. It draws on examples from 24 EbA projects in French territories and highlights a broad spectrum of barriers. The summary analytics of the projects are nicely presented. The broad topic should be of interest to those working in the field of NbS. However, it was unclear to this reader what new knowledge or new critical insights were gleaned from the study? Such contributions need to be heightened in the revision.

The study focusses on barriers to implementation, and the large majority of these barriers are similar to other environmental management settings (coastal, terrestrial ecosystems, rivers). Line 290 even states these findings are known. So what new knowledge is revealed in the analysis? The manuscript does not engage with these broader literatures in a substantive manner. What differentiates the findings of this study from those in these other contexts? Is it the climate change dimension – though other systems are also grappling with similar issues. Reflecting on such similarities and differences in the discussion may strengthen the manuscript. Without a better articulation of this novelty the manuscript largely reads like a list of barriers that are well known.

Following are some further comments on the manuscript structure that the author should consider in a revision.

1. The introduction is a dense list of definitions. Perhaps the definitions could be tabulated, therefore allowing space to develop the intellectual argument of the paper.

2. The section that introduces the case studies is similarly a dense list of percentages and categories. Could these also be tabulated allowing a broader reflection on the purpose of the studies and range of unique small island projects? Also, the manuscript does not reveal whether case studies were successful. How many were considered successful? If none, what does this say about Eba approaches.

3. The author has produced a number of succinct well-presented figures that summarise the broad results. However, in providing high level summaries the reader is not presented with detailed evidence or nuances of the case studies. Furthermore, as little detail is presented, in multiple places conclusions are stated without clear evidence to support them. For example, in lines 358-369 causal mechanisms are asserted but data supporting these statements is not transparent. This is unfortunate as the study clearly has a rich dataset to draw on. Without such ground-truthed details the manuscript reads as a rather generic summary. Can a number of more detailed examples be used to illustrate the main messages?

4. As explicitly outlined in the manuscript the dataset are drawn exclusively from French territories. Are there any unique aspects of this selection that are worthy of highlighting? Are there some findings that would be less relevant in other locations?

# Editor report: Recommendation: Assessing barriers to ecosystem-based adaptation: Application to tropical islands — R1/PR9

**DOI:** 10.1017/cft.2026.10035.pr9

**Affiliations:** Institute for Coastal and Marine Research, Nelson Mandela University, South Africa

The two reviewers provided some valuable input that will improve the manuscript.

From my side please add a metadata sheet to the supplementary excel files stating name of article, contact details, briefly how the data were collected.

# Editor report: Decision: Assessing barriers to ecosystem-based adaptation: Application to tropical islands — R1/PR10

**DOI:** 10.1017/cft.2026.10035.pr10

**Affiliations:** Geography, Cambridge University, United Kingdom of Great Britain and Northern Ireland

No accompanying comment.

# Editor report: Recommendation: Assessing barriers to ecosystem-based adaptation: Application to tropical islands — R2/PR12

**DOI:** 10.1017/cft.2026.10035.pr12

**Affiliations:** Institute for Coastal and Marine Research, Nelson Mandela University, South Africa

THE EDITORIAL OFFICE CAN MAKE THE FOLLOWING CHANGES IF THE AUTHOR AGREES

Please see suggested change to the title of Figure 1 - if the author agrees this change can be made by editorial office

Figure 1. Mapping of study Ecosystem-based Adaptation (EbA) projects with the specific type of intervention undertaken. These maps highlight the location and nature of EbA projects with the various interventions implemented (refer to icon key for interventions) in the five study French overseas tropical island territories. Projects are classified by EbA type (1 to 3 in the legend), and technical actions are described.

AUTHOR TO PROVIDE REVISED EXCEL FILE The excel file - SM4 DATABASE - should have a front sheet with metadata - title of article, author contact details, details on what each of the sheets contain

OTHER EDITS

Line 67: In the introduction, the first sentence, please insert approaches/initiatives after “ecosystem-based” 

Line 86: should be “remove” instead of “removing” 

# Editor report: Decision: Assessing barriers to ecosystem-based adaptation: Application to tropical islands — R2/PR13

**DOI:** 10.1017/cft.2026.10035.pr13

**Affiliations:** Geography, Cambridge University, United Kingdom of Great Britain and Northern Ireland

No accompanying comment.
